# Deactivation Modes in Nickel-Mediated Suzuki–Miyaura Cross-Coupling Reactions Using an NHC-Pyridonate Ligand

**DOI:** 10.1021/acs.organomet.4c00235

**Published:** 2024-08-10

**Authors:** Abhishek A. Kadam, Medina Afandiyeva, William W. Brennessel, C. Rose Kennedy

**Affiliations:** Department of Chemistry, University of Rochester, Rochester, New York 14627, United States

## Abstract

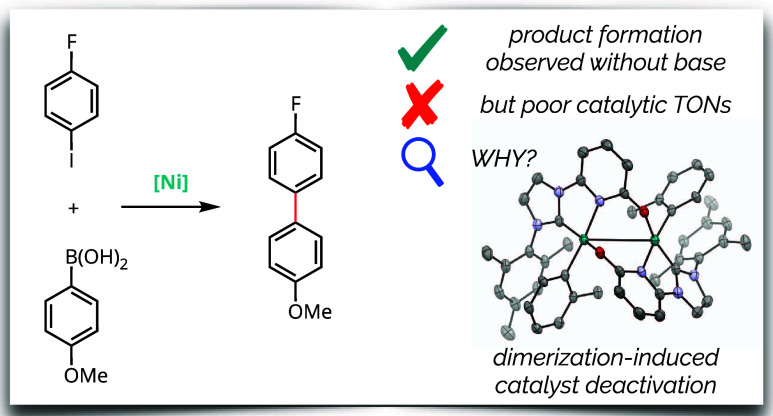

The catalytic activity of an NHC-pyridonate-supported nickel(0) complex for Suzuki–Miyaura coupling of aryl halides was evaluated. Product formation was observed in the absence of a basic additive. However, low turnover numbers resulted from competitive catalyst deactivation. The nature of deactivation—dimerization of the nickel(II) aryl intermediate—was elucidated through a combination of NMR monitoring, direct synthesis, and X-ray diffraction. Lewis basic and Lewis acidic additives were evaluated with the goal of improving the stability of the nickel(II) aryl intermediate but failed to enable catalytic turnover. Taken together, these findings highlight both the promise and the pitfalls associated with incorporating secondary-sphere Lewis basic groups for cooperative catalysis.

## Introduction

**Scheme 1:**
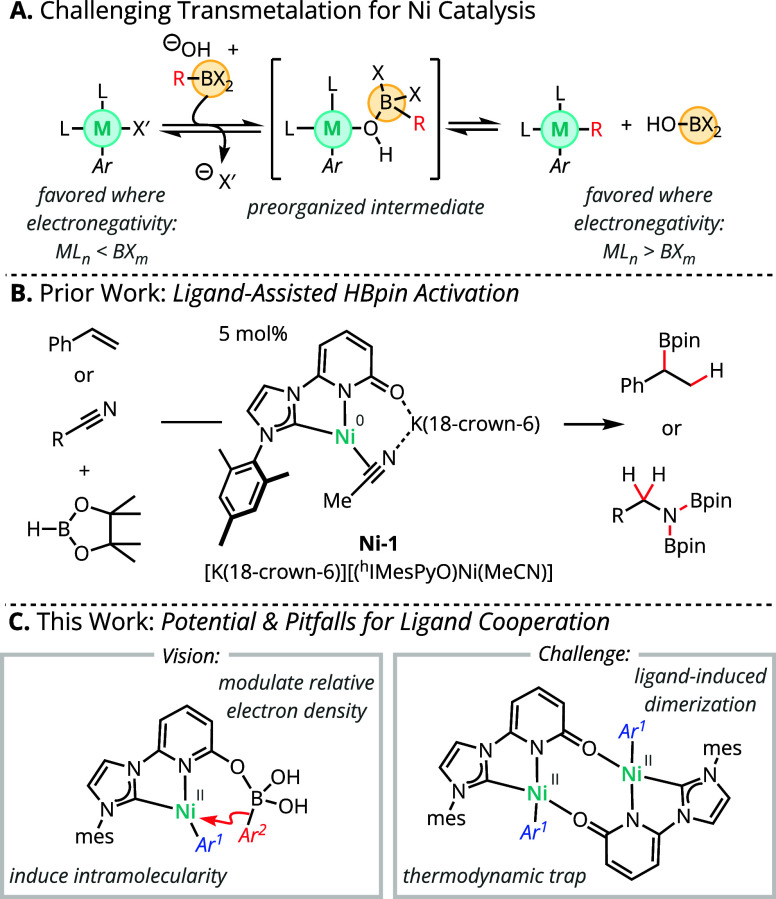
Context and Design Strategy for Suzuki–Miyaura Cross Coupling Using NHC-Pyridonate-Supported Nickel

Suzuki–Miyaura cross-coupling reactions between (typically) aryl halide electrophiles and organoboron nucleophiles have become among the most widely adopted synthetic transformations in fields spanning medicinal chemistry, agrochemical manufacturing, and synthesis of organic electronic materials.^1−8^ The utility of this transformation stems from the bench stability and ease-of-handling typical of boronic acid nucleophiles along with the reliability of highly optimized palladium catalyst systems informed by decades of mechanistic study.^9−12^ Despite these appealing features, there is substantial interest in the development of alternative catalytic methods using more terrestrially abundant metals such as nickel.^13,14^ Notwithstanding promising successes, there remain substantial challenges to the development of nickel-catalyzed Suzuki–Miyaura cross-coupling reactions that rival their palladium-catalyzed counterparts.^13,15−17^ One such limitation is the comparatively unfavorable transmetalation of organoboron nucleophiles to nickel vs palladium (Scheme 1A).^18−20^ This arises, in part, due to the lower electronegativity of nickel (χ_P_ = 1.91) compared to palladium (χ_P_ = 2.20) and boron (χ_P_ = 2.04).^21,22^ As transmetalation equilibria generally favor aryl or alkyl transfer from a more electron-rich (or less electronegative) to a less electron-rich (or more electronegative) metal or metalloid, strong activators are generally required to modulate the relative electron densities at nickel and boron.

Our group previously reported NHC-pyridonate-supported (NHC = N-heterocyclic carbene) nickel complexes that enable secondary-sphere activation of HBpin (4,4,5,5-tetramethyl-1,3,2-dioxaborolane) for hydroboration of alkenes and nitriles (Scheme 1B).^23,24^ NHC ligands are frequently employed in a variety of nickel-catalyzed cross-coupling reactions.^16,25−27^ Accordingly, we envisioned a similar secondary-sphere activation mode may be accessible for organoboron reagents in the context of Suzuki–Miyaura coupling, wherein association of the Lewis basic pyridonate oxygen and Lewis acidic organoboron reagent would simultaneously modulate the relative electron richness of both transmetalation partners and provide rate acceleration through induced intramolecularity (Scheme 1C). In principle, this approach could obviate the need for a basic hydroxide or alkoxide activator.^19,20^ During our studies, a conceptually similar strategy using a phosphine ligand bearing a tethered alcohol was reported by Diao and co-workers.^28^

Herein we describe efforts toward the development of a nickel-catalyzed Suzuki–Miyaura cross-coupling reaction utilizing an NHC-pyridonate ligand to support secondary-coordination-sphere nucleophile activation (Scheme 1C). Through the combination of catalytic screening, NMR monitoring, direct synthesis, and structure elucidation through single-crystal X-ray diffraction (SC-XRD) analysis, these studies expose an ancillary-ligand-mediated mechanism for catalyst deactivation in the presence of aryl halide substrates. A hypothesis-driven selection of additives is evaluated to interrupt the mechanism of nickel deactivation with limited success. This work thus highlights both the promise and the pitfalls associated with leveraging secondary-sphere incorporation of Lewis basic groups for catalysis.

## Results and Discussion

**Scheme 2:**
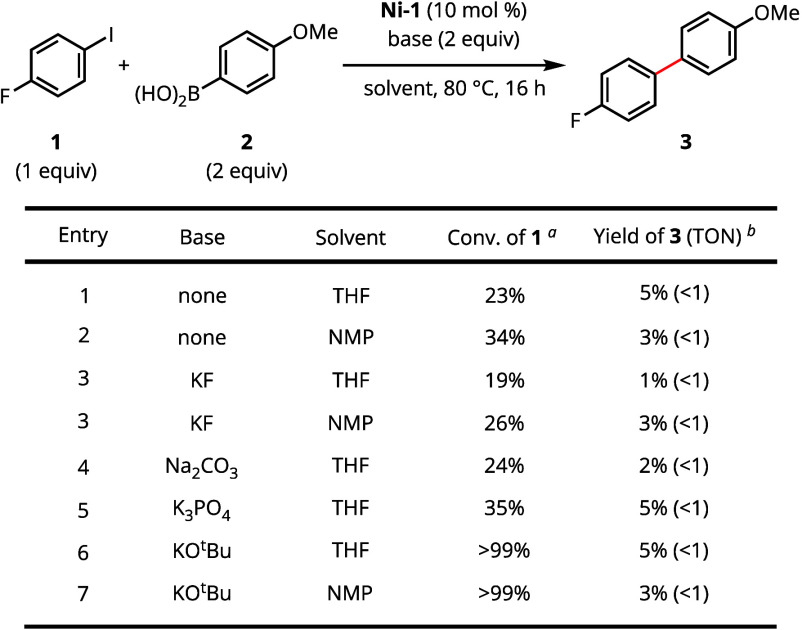
Initial Survey of Catalytic Activity in a Model Suzuki–Miyaura Cross-Coupling Reaction Determined by GC from integration relative to dodecane as an internal standard. Based on ^1^H and ^19^F NMR integration of diagnostic resonances relative to dodecane and fluorobenzene as internal standards.

For evaluation of Suzuki–Miyaura cross-coupling catalysis, we selected 4-fluoroiodobenzene (**1**) and 4-(methoxy)phenylboronic acid (**2**) as model substrates, due to their diagnostic ^19^F and ^1^H NMR signatures. An initial survey of reaction conditions is summarized in Scheme 2; a complete collection of conditions evaluated is provided in the Supporting Information (Tables S1–S5).

Using 10 mol % of [K(18-crown-6)][(^h^IMesPyO)Ni(MeCN)] (**Ni-1**, where ^h^IMesPyO = 1-(2,4,6-trimethylphenyl)-3-(6-oxidopyridin-2-yl)-imidazol-2-ylidene) in either the absence or presence of base afforded biaryl **3** in 3–5% yield after 16 h at 80 °C in THF. Neither the inclusion nor identity of base had a substantive impact on the overall yield of cross-coupled product **3**. However, use of KO^t^Bu as base resulted in substantially increased levels of protodehalogenation as well as both electrophile and nucleophile homocoupling (see Supporting Information, Table S2). While arene solvent (toluene) inhibited reactivity, presumably due to low solubility, other Lewis basic solvents (e.g., NMP, 1,4-dioxane) performed similarly to THF.

The nonzero product yield in the presence of **Ni-1** and absence of base indicates that (i) an exogenous activator is not necessary to achieve transmetalation of boronic acid **2** and (ii) the active complex is capable of productive C–C bond formation. However, the low turnover numbers (TON < 1) suggest that some deactivation process limits regeneration of an active catalyst. We recognized that this deactivation process could result either (i) from failure to regenerate the active complex in the first catalytic turnover or (ii) from a competitive mechanism for catalyst death at another point along the purported catalytic cycle.

**Scheme 3:**
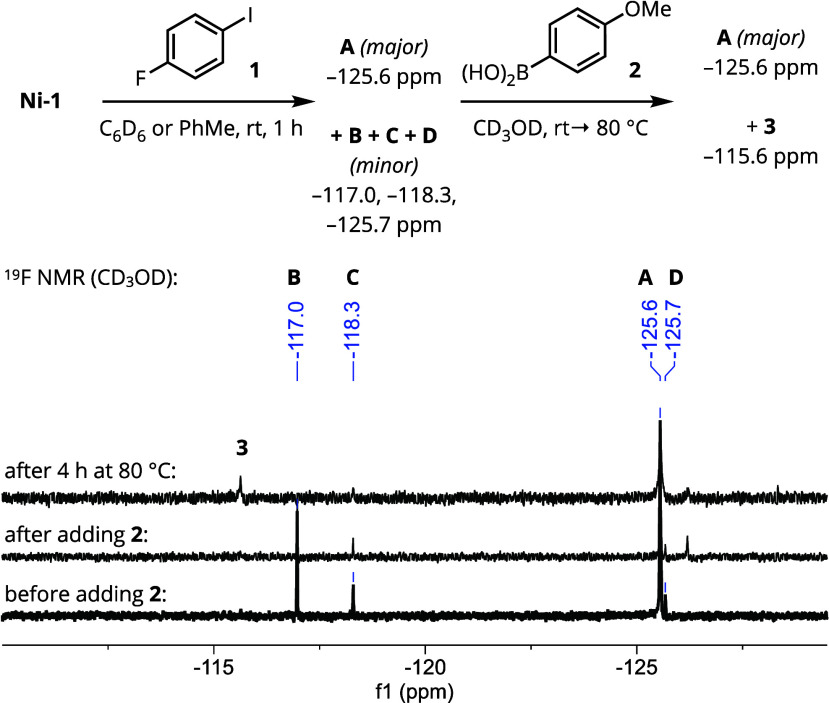
^19^F NMR Monitoring of Nickel Speciation

We envisioned that monitoring the fate of **Ni-1** and electrophile **1** by ^1^H and ^19^F NMR spectroscopy would prove to be informative in evaluating these possibilities. However, upon treatment of **Ni-1** with electrophile **1** (1 equiv) in benzene-*d*_6_ or toluene, the resulting product precipitated from solution, precluding further analysis. Following a survey of routinely available deuterated solvents, methanol-*d*_4_ was identified as affording the highest quality spectra (balancing solubility, stability, and cost) for both **Ni-1** and its resulting products. Upon resuspending the crude precipitate in methanol-*d*_4_, ^1^H and ^19^F NMR spectroscopic features were found to be consistent with a single major (^19^F NMR δ: −125.6 ppm) and three minor (^19^F NMR δ: −117.0, −118.3, and −125.7 ppm) fluorophenyl-containing products (Scheme 3, Figures S12–S13). Upon the addition of boronic acid **2** and heating to 80 °C for 4 h, the three minor species were largely consumed, as evidenced by the disappearance or decreased magnitude of the ^19^F NMR resonances at −117.0, – 118.3, and −125.7 ppm, coincident with the appearance of a new signature at −115.6 ppm (corresponding to product **3**). However, the resonance at −125.6 ppm persisted, even after heating to 80 °C for 24 h (see Supporting Information, Figures S16–S17). These observations suggested that understanding the structure and identity of this major component would be crucial for understanding the deactivation processes preventing catalytic turnover.

Purification and complete structural elucidation of the products derived from electrophile **1** proved to be challenging. We therefore elected to use 2-iodotoluene (**4a**) and 2-bromotoluene (**4b**) as model electrophiles for isolation experiments due to the precedent for ortho-substitution stabilizing otherwise challenging-to-isolate Ni(II) aryl complexes.^29−33^ To enable comparison, a series of experiments were performed in which excess 2-halotoluene (**4a** or **4b**) was added at ambient temperature to a solution of either anionic **Ni-1** or neutral analogue **Ni-2**, which lacks an O substituent (**Ni-2** = [(^h^IMesPy)Ni(cod)], where ^h^IMesPy = 1-(2,4,6-trimethylphenyl)-3-(2-pyridyl)-imidazol-2-ylidene).^34^ In each case, a color change from purple to crimson red or orange was observed within 1–2 h. The resulting suspensions were concentrated in vacuo and washed with pentane and diethyl ether prior to analysis by ^1^H NMR spectroscopy and recrystallization from THF to afford crystals suitable for SC-XRD.

**Scheme 4:**
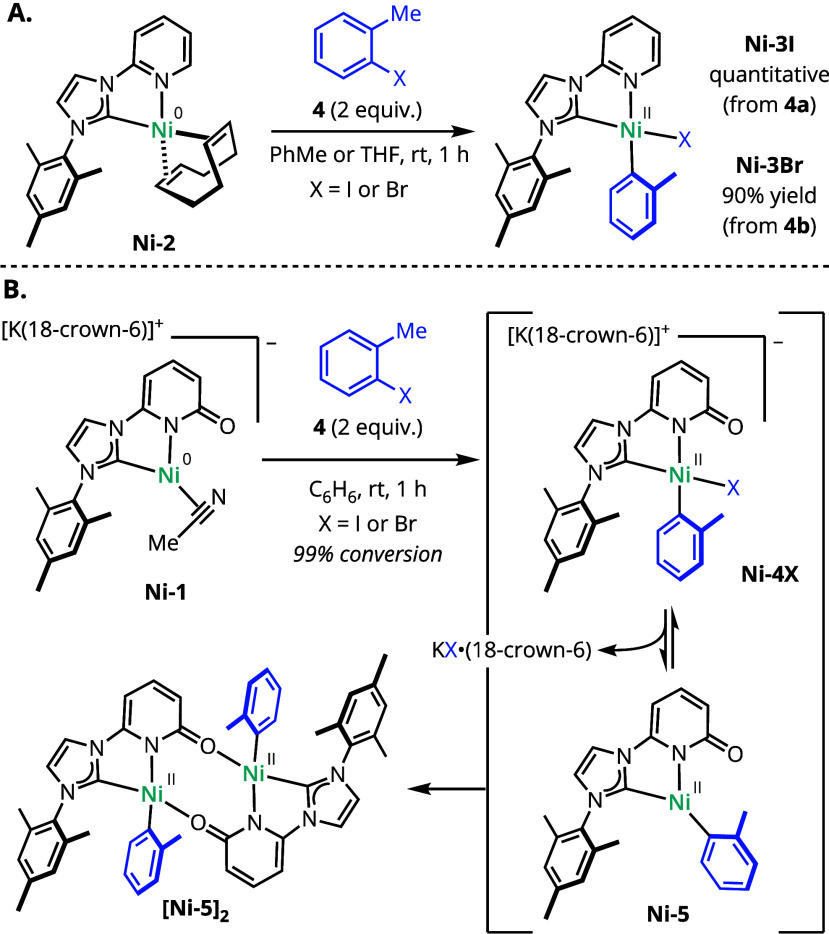
Oxidative Addition Reactivity of NHC-Pyridine and NHC-Pyridonate-Supported Ni Complexes

As expected, **Ni-2** reacted with 2-halotoluenes **4a**–**b** to afford the corresponding classical oxidative addition products, [(^h^IMesPy)NiX(*o*-tol)], where X = I (**Ni-3I**) or Br (**Ni-3Br**) (Scheme 4A).^35^ The solid-state coordinates further revealed the exclusive formation of the isomer with the electron-rich, σ-donating aryl ligand trans to the π-accepting pyridine. By contrast, **Ni-1** reacted with either **4a** or **4b** in C_6_H_6_ to afford the same diamagnetic product in both cases (Scheme 4B). The analogous reaction with 2-chlorotoluene (**4c**) required extended time and elevated temperature (50 °C, overnight) but also yielded the same diamagnetic product (see Supporting Information, Section 3.3). These results indicated that the halide was not retained in the final structure and the relative coordinating abilities of I^–^, Br^–^, and Cl^–^ did not substantially impact the reaction. Single-crystal X-ray diffraction (SC-XRD) analysis revealed the structure of neutral, dimeric Ni(II) aryl complex **[Ni-5]**_**2**_. Presumably, this dimeric adduct formed following oxidative addition and dissociation of the halide ligand to afford three-coordinate Ni(II) complex **Ni-5**. In the absence of an alternative neutral ligand, the pyridonate acted as a 1,3-bridging ligand. Consistent with this hypothesis, colorless crystals of [K(18-crown-6)]I were obtained from the crude reaction mixture, and their composition was validated by SC-XRD.

**Figure 1:**
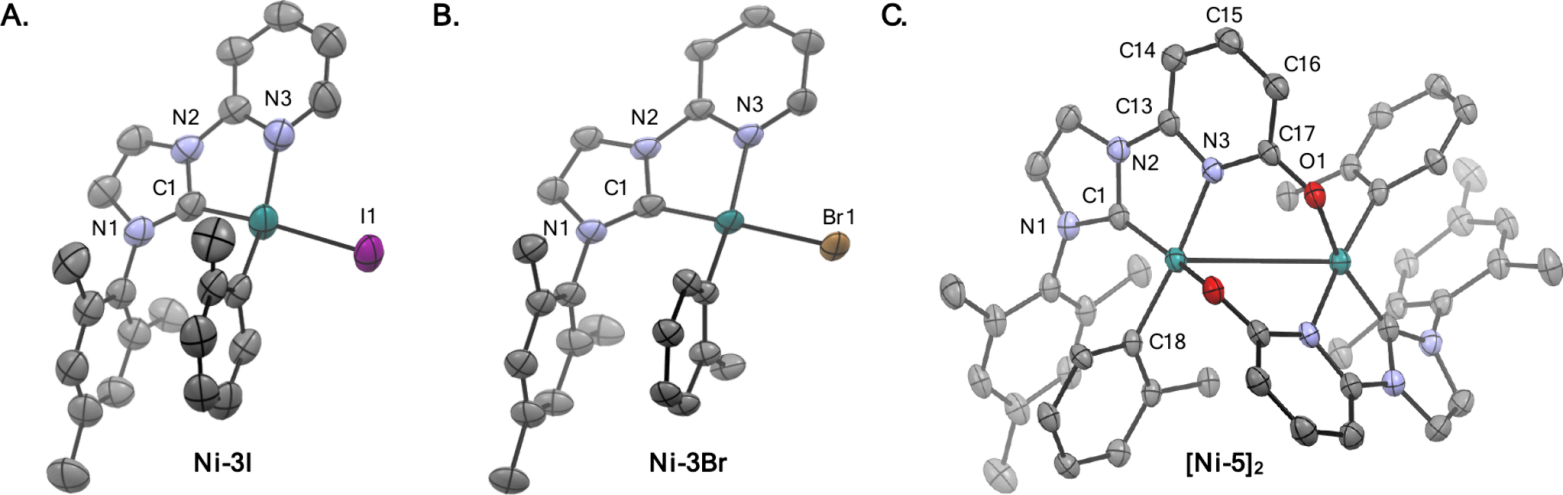
Solid-state structures of (A) **Ni-3I**, (B) **Ni-3Br**, and (C) **[Ni-5]**_**2**_ determined by single-crystal X-ray diffraction. Thermal ellipsoids are depicted at 50% probability. H atoms and cocrystallized solvent molecules are omitted for clarity. Only one of two stereoisomers arising from disorder of the tolyl ligand over two positions shown for **Ni-3I** (0.54:0.46), **Ni-3Br** (0.62:0.38), and **[Ni-5]**_**2**_ (0.82:0.18). C = charcoal, N = blue, O = red, Ni = teal, Br = bronze, I = magenta.

The solid-state structures and key bonding metrics for **Ni-3I**, **Ni-3Br**, and **[Ni-5]**_**2**_ are shown in Figure 1 and Table 1. Most notably, the (pyridonate)N–Ni and (pyridonate)O–Ni bond lengths were found to be 1.9755(19) and 1.9187(16) Å, and the bond length of the pyridonate C–O bond was found to be 1.279(3) Å, suggesting double-bond character. Taken together with the alternating bond lengths of the pyridonate backbone, these metrics are most consistent with the pyridonate ligand serving as a L_O_,X_N_ type ligand.^36−38^ This stands in contrast to the L_N_,X_X_ coordination observed with classical products **Ni-3I** and **Ni-3Br**.

**Table 1:** Key Metrics for the Solid-State Structures of **Ni-3I**, **Ni-3Br**, and **[Ni-5]_2_** Determined by SC-XRD

bond lengths (Å) and dihedral angles (deg)				bond angles (deg)			
	**Ni-3I**	**Ni-3Br**	**[Ni-5]**_**2**_		**Ni-3I**	**Ni-3Br**	**[Ni-5]**_**2**_
Ni(1)–X	2.503(5)	2.3341(6)	1.9187(16)	C(1)–Ni(1)–X	163.8(8)	168.39(9)	159.06(8)
	2.550(4)				169.0(7)		
Ni(1)–N(3)	2.016(6)	2.008(2)	1.9755(19)	C(1)–Ni(1)–N(3)	83.2(2)	82.63(11)	82.29(9)
	2.005(6)				82.4(3)		
Ni(1)–C(1)	1.845(6)	1.867(3)	1.857(2)	C(1)–Ni(1)–C(18)	94.1(8)	96.9(4)	95.39(10)
	1.891(7)				95.8(9)	91.2(6)	93.4(3)
Ni(1)–C(18)	1.911(8)	1.906(5)	1.910(3)	C(18)–Ni(1)–X	87.0(8)	88.5(4)	86.79(9)
	1.900(8)	1.914(7)	1.914(8)		90.5(9)	92.3(6)	98.4(3)
N(3)–C(13)	1.337(6)	1.340(4)	1.351(3)	C(18)–Ni(1)–N(3)	170.2(10)	166.3(3)	173.70(10)
N(3)–C(17)	1.344(7)	1.338(4)	1.359(3)		166.1(10)	172.7(6)	156.3(3)
C(13)–C(14)	1.380(6)	1.390(3)	1.364(3)	N(3)–Ni(1)–X	98.2(2)	94.50(8)	93.30(7)
C(14)–C(15)	1.379(7)	1.380(5)	1.401(4)		93.7(2)		
C(15)–C(16)	1.370(8)	1.377(5)	1.371(4)	N(1)–C(1)–N(2)	103.1(4)	102.9(2)	103.37(19)
C(16)–C(17)	1.377(8)	1.389(4)	1.423(3)	C(13)–N(3)–C(17)	116.6(4)	117.9(2)	119.00(19)
O(1)–C(17)			1.279(3)	N(3)–C(13)–C(14)	124.2(5)	123.9(3)	125.0(2)
				C(13)–C(14)–C(15)	117.7(5)	117.0(3)	116.1(2)
C(1)–N(2)–C(13)–N(3)	2.7(6)	1.1(4)	3.9(3)	C(14)–C(15)–C(16)	119.4(5)	120.1(3)	120.7(2)
C(1)–N(2)–C(13)–C(14)	–176.8(4)	–178.2(3)	–177.7(2)	C(15)–C(16)–C(17)	119.0(6)	118.9(3)	119.9(2)
				C(17)–O(1)–Ni(1′)			125.65(14)
				N(3)–C(17)–O(1)			119.7(2)

Comparing the ^1^H NMR spectrum obtained following the reaction of **Ni-1** and **1 with those** of **Ni-3I**, **Ni-3Br**, and **[Ni-5]**_**2**_ reveals a high degree of similarity with **[Ni-5]**_**2**_, consistent with an analogous dimeric structure accounting for persistent major product **A** (see Supporting Information, Figures S14–S15). This assignment suggests that dimerization of key oxidative addition intermediates introduces a thermodynamic trap, thereby accounting for the low TONs observed under catalytic conditions. Consistent with this hypothesis, **[Ni-5]**_**2**_ is ineffective as a precatalyst, leading to no consumption of **1** under standard conditions (see Supporting Information, Table S2).

**Scheme 5:**
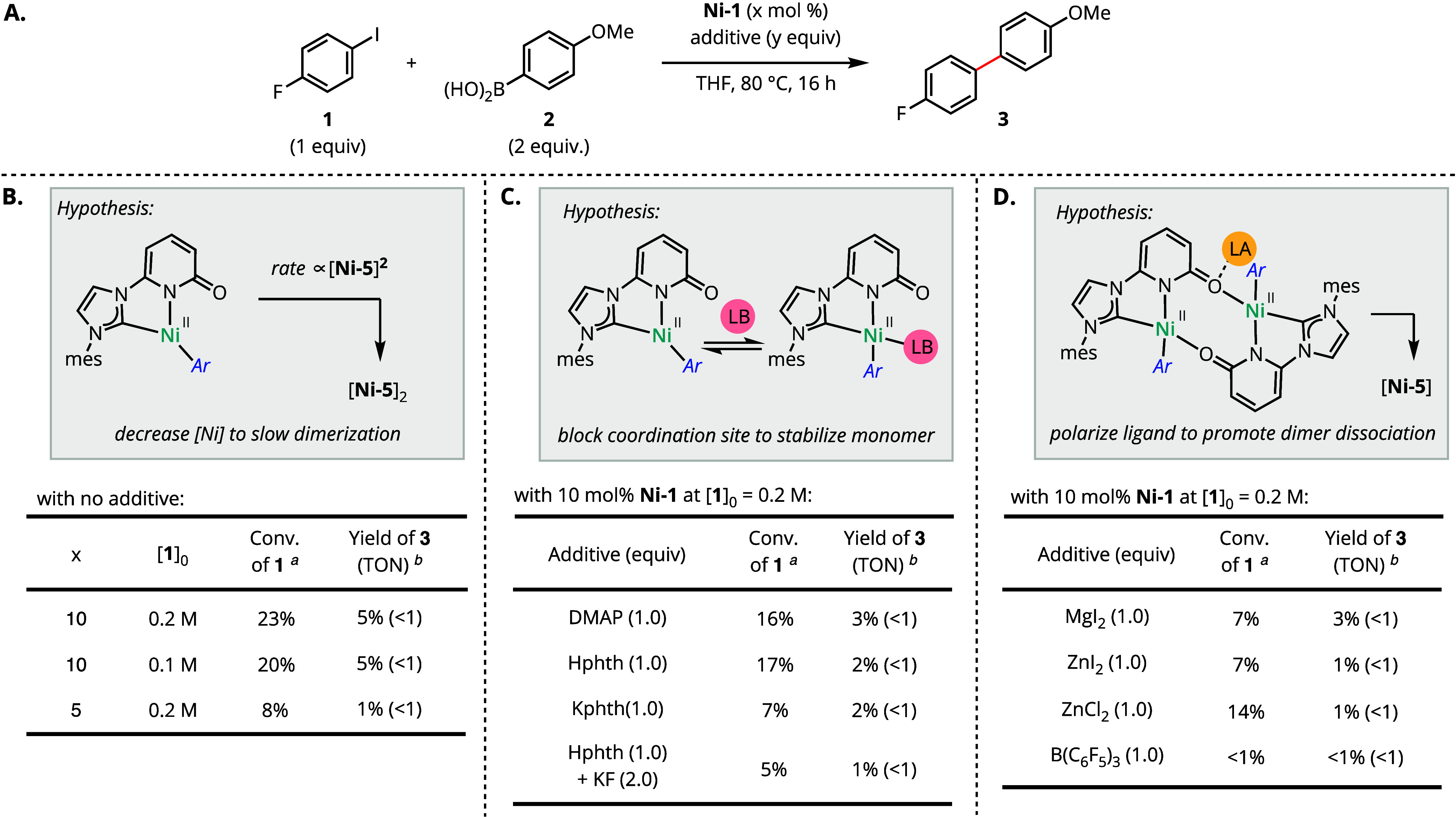
Hypothesis-Driven Modification of Reaction Conditions to Address Catalyst Deactivation Determined by gas chromatography from integration relative to dodecane as an internal standard. ^*b*^ Based on ^1^H and ^19^F NMR integration of diagnostic resonances relative to dodecane and fluorobenzene as internal standards.

Nonetheless, identification of the catalyst deactivation pathway informed strategies to address the limitation by either preventing dimerization or providing a path for dimer dissociation (Scheme 5). Recognizing the bimolecular kinetic dependence of catalyst dimerization, we first assessed the impact of nickel concentration on efficiency. However, neither decreasing the global concentration nor reducing the loading of **Ni-1** resulted in any improvements to the TON or yield of biaryl **3** (Scheme 5B). Lewis basic additives were assessed with the goal of temporarily blocking the vacant coordination site in purported monomeric intermediate **Ni-5** (Scheme 5C).^39^ However, the added Lewis bases were insufficient to overcome this challenge. Lewis acid additives were also evaluated with the goal of promoting dissociation of [**Ni-5**]_2_ through competitive coordination with the pyridonate O (Scheme 5D). However, neither Zn^2+^ or Mg^2+^ salts resulted in improved cross-coupling yields. Introduction of B(C_6_F_5_)_3_ inhibited cross coupling altogether and afforded an intractable mixture of products.

## Conclusion

In summary, a bifunctional NHC-pyridonate-supported nickel complex showed activity for Suzuki–Miyaura coupling in the absence of exogenous base additives but was limited by poor turnover. Mechanistic study enabled identification of a unique catalyst deactivation pathway involving ligand-promoted dimerization. These results thus highlight challenges associated with ligand designs leveraging potentially reactive sites in the secondary coordination sphere. While additives proved to have limited beneficial effect in this case, elucidation of the ancillary-ligand-induced deactivation pathway informs future design strategies to overcome these challenges. Work toward this aim is ongoing in our research group and will be reported in due course.

## Experimental Section

### General Experimental Details

All air- and moisture-sensitive techniques were carried out using standard Schlenk technique on a high-vacuum line^40^ or in an M. Braun glovebox containing an atmosphere of N_2_. The glovebox was equipped with vacuum feed-throughs, a cold well, and a freezer for storing samples at −30 °C. Additional details are provided in the Supporting Information.

### General Procedure for Catalytic Screening

In an N_2_-filled glovebox, a 1-dram vial equipped with a PTFE-coated stir bar was charged with nickel complex **Ni-1** (0.014 g, 0.010 mmol, 10 mol %), 4-methoxyboronic acid (**2**, 0.061 g, 0.4 mmol, 2.0 equiv), additive (0.02–0.4 mmol, 0.2–2.0 equiv) and then 4-fluoroiodobenzene (**1**, 23 μL, 0.2 mmol, 1.0 equiv) and solvent (1.0 mL). The vial was capped and sealed with electrical tape, removed from the glovebox, and stirred at 80 °C for 16 h. The vial was then opened to air, dodecane (20 μL) was added as an internal standard, and an aliquot was removed for gas chromatographic analysis. The crude reaction mixture was passed through silica and eluted with 7:3 hexanes/ethyl acetate (15 mL). The filtrate was concentrated in vacuo and then dissolved in CDCl_3_ for analysis by ^19^F and ^1^H NMR spectroscopy using fluorobenzene or dibromomethane as internal standards, respectively.

### Oxidative Addition Product of **Ni-2** and 2-Iodotoluene (**Ni-3I**)

In an N_2_-filled glovebox, **Ni-2** (50.0 mg, 0.116 mmol, 1.00 equiv) was weighed into a scintillation vial. The scintillation vial was then charged with 2-iodotoluene (50.7 mg, 29.6 μL, 0.232 mmol, 2.00 equiv) and toluene (3 mL). The resulting yellowed orange solution was stirred vigorously at room temperature for 1 h. The reaction mixture was then concentrated in vacuo to obtain a crimson red solid. A monster pipet was loaded with a short pad of Celite (approximately 2 cm height), and the Celite was packed with pentane. The crimson red solid was then resuspended in pentane and passed through Celite rinsing with a total of ∼10 mL of pentane total (colorless solution). The crimson red solid left on top of the Celite was further rinsed with a total of ∼10 mL of diether ether (light orange solution). The ether solution was discarded. The red solid was then rinsed with THF until colorless solution started eluting. The THF solution was concentrated in vacuo to obtain a crimson red solid in quantitative yield (0.0663 g). To obtain single crystals suitable for XRD, the product was dissolved in a minimal amount of THF in a 1-dram vial. The 1-dram vial was placed in a scintillation vial containing pentane. The scintillation vial was sealed and left at room temperature.

### Oxidative Addition Product of **Ni-2** and 2-Bromotoluene (**Ni-3Br**)

In an N_2_-filled glovebox, **Ni-2** (50.0 mg, 0.116 mmol, 1.00 equiv) was weighed into a scintillation vial. The scintillation vial was then charged with 2-bromotoluene (39.7 mg, 27.9 μL, 0.232 mmol, 2.00 equiv) and THF (3 mL). The resulting mixture was stirred vigorously at room temperature for 1 h. After 1 h, the reaction mixture was concentrated in vacuo. A monster pipet was loaded with a short pad of Celite (approximately 2 cm height), and the Celite was packed with pentane. The yellowed orange solid was then resuspended in pentane and passed through Celite rinsing with a total of ∼10 mL of pentane total (colorless solution). The yellowed orange solid left on top of the Celite was further rinsed with a total of ∼10 mL of diether ether (light orange solution). The ether solution was discarded. The yellowed orange solid was then rinsed with THF until a colorless solution started eluting. The THF solution was concentrated in vacuo to obtain a crimson red solid in 90% yield (0.0514 g). To obtain single crystals suitable for XRD, the product was dissolved in a minimal amount of THF, layered with pentane, and left at room temperature. The supernatant was decanted, and the solid was dried in vacuo to obtain a metallic gold/yellow solid in 62% recrystallized yield (0.0356 g).

### Oxidative Addition Product of **Ni-1** and 2-Halotoluene (**[Ni-5]_2_**)

In a N_2_-filled glovebox, **Ni-1** (0.0293 g, 0.043 mmol, 1.00 equiv) was weighed into a scintillation vial. C_6_H_6_ (0.02 M, 2.1 mL) was added to the vial, followed by 2-iodotoluene (10.9 μL, 0.0147 g, 0.086 mmol, 2.00 equiv) or 2-bromotoluene (10.3 μL, 0.0187 g, 0.086 mmol, 2.00 equiv). The resulting mixture was stirred vigorously at room temperature for 1 h. After 1 h, the yellowed orange turbid solution was loaded onto a monster pipet filled with a short pad of Celite (approximately 2 cm height) and passed through Celite rinsing with C_6_H_6_ until the filtrate was colorless. The filtrate was concentrated in vacuo and washed with pentane (2 × 2 mL), decanting the pentane with a pipet after each wash. The solid was dried in vacuo to afford 20.8 mg of the material. To obtain single crystals suitable for XRD, the product was dissolved in a minimal amount of THF, layered with pentane, and left at room temperature.

## Data Availability

An initial draft of this manuscript was posted on ChemRxiv.^41^

## Supporting Information Available

Experimental details, compound characterization data, and supplementary results (PDF)
