# Supplementary material for: Deactivation Modes in Nickel-Mediated Suzuki–Miyaura Cross-Coupling Reactions Using an NHC-Pyridonate Ligand
Source: Organometallics. 2024 Aug 10;43(20):2574–80. doi: 10.1021/acs.organomet.4c00235 (PMC11523216; doi:10.1021/acs.organomet.4c00235)
Supplement: Supplementary file 1 — om4c00235_si_001.pdf [file om4c00235_si_001.pdf]

Supporting Information

Deactivation Modes in Nickel-Mediated Suzuki–Miyaura Cross-Coupling Reactions Using an NHC-Pyridonate Ligand

Abhishek A. Kadam,[‡] Medina Afandiyeva,[‡] William W. Brennessel, and C. Rose Kennedy*

Department of Chemistry, University of Rochester

Rochester, New York 14627, United States

‡ Equal authorship

*Corresponding Author: c.r.kennedy@rochester.edu

Table of Contents for the Supporting Information

1. Procedures, Materials, and Instrumentation S3

1.1 General Considerations S3

1.2 Materials S3

1.3 Instrumentation and Software S3

1.4 Abbreviations S4

2. Summary of Catalytic Screens S5

2.1 General procedure for catalytic screens S5

2.2 Evaluation of base, solvent, & temperature using **Ni-1** as precatalyst S6

2.3 Control experiments using **Ni-2** and [**Ni-5**]$_2$ as precatalysts S6

2.4 Evaluation of additives using **Ni-1** as precatalyst S8

2.5 Evaluation of alternative electrophiles using **Ni-1** as precatalyst S8

3. Synthesis & Characterization of Nickel Complexes S10

3.1 Oxidative Addition Product of **Ni-2** and 2-Iodotoluene (**Ni-3I**) S10

3.2 Oxidative Addition Product of **Ni-2** and 2-Bromotoluene (**Ni-3Br**) S13

3.3 Oxidative Addition Product of **Ni-1** and 2-Halotoluene (**[Ni-5]$_2$**) S16

3.4 Oxidative Addition Product of **Ni-1** and 4-Fluoroiodobenzene S19

3.5 Summary of Crystallographic Data and Refinement Details S22

4. Mechanistic Experiments S23

4.1 ^{19}F NMR Monitoring in Methanol-*d*$_4$ S23

4.2 ^{19}F NMR Monitoring in THF-*d*$_8$ S27

5. References S29

1. Procedures, Materials, and Instrumentation

1.1 General Considerations

All air- and moisture-sensitive techniques were carried out using standard Schlenk technique on a Schlenk line or a high-vacuum line or in an M. Braun glovebox containing an atmosphere of N_2. The glovebox was equipped with vacuum feed-throughs, a cold well, and a freezer for storing samples at –30 °C. Colors are described in comparison to the complete list of Prismacolor colored pencils. Column chromatography was performed on SiliaFlash P60 (230–400 mesh) silica gel from SiliCycle using standard glass columns. Thin-layer chromatography (TLC) was performed using aluminum-backed plates pre-coated with silica gel and a fluorescent indicator for visualization upon UV irradiation.

1.2 Materials

Reagents were purchased in reagent grade from commercial suppliers and used without further purification unless described otherwise. Liquid haloarenes were stirred over and then distilled from calcium hydride prior to use. Boronic acids, ZnI_2, phthalimide, and *N,N*-dimethylaminopyridine (DMAP) were dried under vacuum overnight and transferred to the glovebox for storage and use. Bis(cyclooctadiene) nickel [$Ni(cod)_2$] was purchased from Strem and stored at –30 °C in the glovebox. Nickel complexes **Ni-1**[1] and **Ni-2**[2] were was prepared as reported previously. Solvents (acetonitrile, diethyl ether, *n*-pentane, tetrahydrofuran, and toluene) used for air- and moisture-sensitive manipulations were dried and deoxygenated by passage through an activated alumina column[3] and stored over activated molecular sieves. Deuterated solvents used for NMR spectroscopy of air- and moisture-sensitive compounds were stirred over sodium (C_6D_6, THF-d_8) or calcium hydride (CD_3CN) and distilled prior to storage in the glovebox. Methanol-d_4 (CD_3OD) was purchased in air-free ampules and used without further purification.

1.3 Instrumentation and Software

Proton nuclear magnetic resonance (^{1}H NMR) spectra were recorded at 25 °C on Bruker 400 or 500 Avance I spectrometers operating at 400.13 or 500.20 MHz, respectively, or on a JEOL 400 (JNM-ECZL S) or 500 (JNM-ECZL R) spectrometers operating at 399.78 or 499.96 MHz, respectively. Proton-decoupled ^{13}C NMR spectra were recorded at 25 °C on a Bruker 500 Avance I spectrometer operating at 125.78 MHz. Proton-decoupled ^{19}F NMR spectra were recorded at 25 °C on a Bruker 400 spectrometer operating at 376.15 MHz. All experiments were performed at the University of Rochester, Department of Chemistry, Nuclear Magnetic Resonance Facility; the JEOL instruments were purchased with funding from NSF MRI program grant CHE-2215973. ^{1}H and ^{13}C chemical shifts are reported in parts per million downfield from tetramethylsilane ($SiMe_4$) and are referenced in ppm relative to the NMR solvent according to literature values:[4] $\delta(^1H)$ = 7.16, $\delta(^{13}C)$ = 128.1 for C_6D_6.; $\delta(^1H)$ = 1.94, $\delta(^{13}C)$ = 118.3 for CD_3CN; $\delta(^1H)$ = 1.72, 3.58, $\delta(^{13}C)$ = 67.2, 25.3 for THF-d_8; $\delta(^1H)$ = 7.26, $\delta(^{13}C)$ = 77.2 for $CDCl_3$; $\delta(^1H)$ = 3.31, $\delta(^{13}C)$ = 49.0 for CD_3OD. ^{19}F chemical shifts are reported in parts per million downfield from neat $CFCl_3$ based up referencing to either fluorobenzene (–112.96 ppm in $CDCl_3$, –113.11 in C_6D_6, −115.42 in CD_3OD) or α,α,α-trifluorotoluene

(–62.61 in $CDCl_3$, –62.74 in C_6D_6, −64.24 in CD_3OD) as an internal standard.[5] ^{1}H NMR data for diamagnetic substances are reported as follows: chemical shift, (multiplicity, coupling constant in Hz, integration) where s = singlet, d = doublet, t = triplet, q = quartet, m = multiplet, and br = broad. ^{13}C and ^{19}F NMR data for diamagnetic substances are reported as a list of chemical shifts. NMR spectra were processed using the MestReNova software suite. Low resolution mass spectra (LRMS) were recorded using a Shimadzu LCMS-2020/2050 system operating with electrospray ionization (ESI) or Shimadzu QP-2010 Gas Chromatography/Mass Spectrometry (GC/MS) instrument operating with electron impact (EI) ionization

Single-crystal X-ray diffraction data collection, structure solution, and structure refinement were conducted at the X-ray Crystallographic Facility, B04 Hutchison Hall, Department of Chemistry, University of Rochester. The Rigaku XtaLAB Synergy-S Dualflex diffractometer equipped with a HyPix-6000HE HPC area detector for data collection at 100.00(10) K was purchased with funding from NSF MRI program grant CHE-1725028. For each crystal, a preliminary set of cell constants and an orientation matrix were calculated from a small sampling of reflections.[6] A short pre-experiment was run, from which an optimal data collection strategy was determined. The full data collection was carried out using a PhotonJet (Cu) X-ray source through a series of frames were collected in 0.50º steps in *w* at different 2*q*, *k*, and *f* settings. After the intensity data were corrected for absorption, the final cell constants were calculated from the xyz centroids of strong reflections from the actual data collection after integration.[6] The structure was solved using SHELXT and refined using SHELXL.[7, 8] The space group was determined based on systematic absences and intensity statistics. Most or all non-hydrogen atoms were assigned from the solution. Full-matrix least squares / difference Fourier cycles were performed which located any remaining non-hydrogen atoms. All non-hydrogen atoms were refined with anisotropic displacement parameters. Unless otherwise noted, all hydrogen atoms were placed in ideal positions and refined as riding atoms with relative isotropic displacement parameters. Structure manipulation and figure generation were performed using Olex2 or Mercury.[9, 10] Unless noted otherwise all structural diagrams containing anisotropic displacement ellipsoids are drawn at the 50 % probability level.

1.4 Abbreviations

Ar = aryl; Bpin = pinacolboryl = 4,4,5,5-tetramethyl-1,3-dioxaboryl; cod = 1,5-cyclooctadiene; DMAP = *N,N*-dimethylpyridine-4-amine; EI = electron impact ionization; ESI = electrospray ionization; ^{h}IMesPyO= 1-(2,4,6-trimethylphenyl)-3-(6-oxidopyridin-2-yl)-imidazol-2-ylidene); LRMS = low-resolution mass spectrometry; NHC = N-heterocyclic carbene; NMP = N-methylpyrrolidinone; NMR = nuclear magnetic resonance spectroscopy; Phthalimide-H = 1*H*-Isoindole-1,3(2*H*)-dione; Phthalimide-K = potassium 1,3-dioxoisoindolin-2-ide; PTFE = poly(tetrafluoroethylene); Py = pyridine; SC-XRD = single-crystal X-ray diffraction; THF = tetrahydrofuran

2. Summary of Catalytic Screens

2.1 General Procedure for Catalytic Screens

Scheme S1. General procedure for catalytic screens.

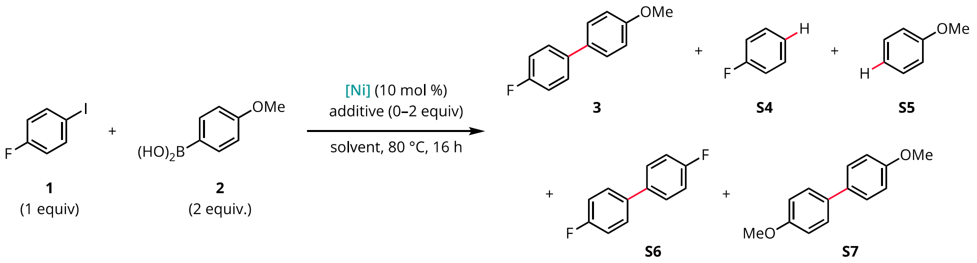

In an N_2-filled glovebox, a 1-dram via equipped with a PTFE coated stir bar was charged with nickel complex (0.010 mmol, 10 mol % unless noted otherwise), 4-methoxyphenylboronic acid (2, 0.061 g, 0.4 mmol, 2.0 equiv), additive (0–0.4 mmol, 0–2.0 equiv), then electrophile (**1**, 23 μL, 0.2 mmol. 1.0 equiv, unless noted otherwise) and solvent (1.0 mL THF, unless noted otherwise). The vial was capped and sealed with electrical tape, removed from the glovebox, and stirred at 80 °C for 16 h. The vial was then opened to air, dodecane (20 μL) was added as an internal standard, and an aliquot was removed for gas chromatographic analysis. The crude reaction mixture was passed through silica and eluted with 7:3 hexanes/ethyl acetate (15 mL). The filtrate was concentrated in vacuo and then dissolved in $CDCl_3$ for analysis by ^{19}F and ^{1}H NMR spectroscopy using fluorobenzene (15.00 μL, 0.1592 mmol) and/or dibromomethane (7.00 μL, 0.100 mmol or 15.00 μL, 0.2157 mmol) as internal standards, respectively.

2.2 Evaluation of base, solvent, & temperature using Ni-1 as precatalyst

Table S1. Evaluation of base, solvent, and temperature using **Ni-1** as precatalyst

Entry	Base	Solvent (M)	Temp (°C)	Conv of **1** (%) [a]	Yield (%)				
					**3** [b]	**S4** [a]	**S5** [a]	**S6** [b]	**S7**
1	none	THF (0.2 M)	80	23%	5%	10%	4%	<1%	n.d.[c]
2	none	THF (0.2 M)	100	15%	2%	16%	12%	<1%	n.d.[c]
3	none	1,4-dioxane (0.2 M)	100	17%	2%	17%	11%	1%	n.d.[c]
4	none	t-amyl alcohol (0.2 M)	100	18%	2%	12%	64%	<1%	n.d.[c]
5	none	NMP (0.2 M)	80	34%	3%	6%	6%	<1%	n.d.[c]
6	none	THF (0.1 M)	80	20%	5%	9%	3%	1%	n.d.[c]
7	KO^{t}Bu	THF (0.2 M)	80	>99%	5%	54%	14%	4%	obs [d]
8	KO^{t}Bu	NMP (0.2 M)	80	>99%	3%	83%	11%	3%	obs [d]
6	KO^{t}Bu	PhMe (0.2 M)	80	>99%	obs [d]	20%	9%	obs [d]	obs [d]
7	KO^{t}Bu	1,4-dioxane (0.2 M)	80	>99%	obs [d]	57%	12%	obs [d]	obs [d]
8	KF	THF (0.2 M)	80	19%	1%	9%	7%	1%	obs [d]
9	KF	NMP (0.2 M)	80	26%	3%	2%	2%	n.d.[c]	n.d.[c]
10	K_3PO_4	THF (0.2 M)	80	35%	5%	6%	13%	10%	n.d.[c]
11	Na_2CO_3	THF (0.2 M)	80	24%	2%	10%	5%	<1%	n.d.[c]

[a] Based on GC integration relative to dodecane; [b] Based on ^{19}F NMR integrations of diagnostic resonances relative to that of fluorobenzene (–113.0 ppm) or α,α,α -trifluorotoluene (–62.6 ppm); [c] Not detected by GC or GC-MS [d] Observed by GC or GC-MS but not quantified due to poor NMR resolution

2.3 Control experiments using Ni-2 and [Ni-5]$_2$ as precatalysts

Table S2. Control experiments using **Ni-2** and **[Ni-5]$_2$** as precatalysts

Entry	[Ni]	Conversion of **1** (%) [a]	Yield (%)				
			**3** [b]	**S4** [a]	**S5** [a]	**S6** [b]	**S7**
1	**Ni-1**	23%	5%	10%	4%	<1%	n.d.[c]
2	**Ni-2**	21%	<1%	4%	1%	<1%	n.d.[d]
3	**[Ni-5]$_2$**	<1%	n.d.[d]	n.d.[d]	4%	n.d.[d]	n.d.[d]

[a] Based on GC integration relative to dodecane; [b] Based on ^{19}F NMR integrations of diagnostic resonances relative to that of fluorobenzene (–113.0 ppm) or α,α,α -trifluorotoluene (–62.6 ppm); [c] Not detected by GC or GC-MS

2.4 Evaluation of additives using Ni-1 as precatalyst

Table S3. Evaluation of additives using **Ni-1** as precatalyst

Entry	Additive	Conversion of **1** (%) [a]	Yield (%)				
			**3** [b]	**S4** [a]	**S5** [a]	**S6** [b]	**S7**
1	none	23%	5%	10%	4%	<1%	n.d.[c]
2	Hphth (1.0 equiv)	17%	2%	8%	9%	<1%	n.d.[c]
3	Hphth (1.0 equiv) + KO^tBu (2.0 equiv)	60%	4%	22%	15%	12%	obs [d]
4	Hphth (1.0 equiv) + KF (2.0 equiv)	5%	<1%	9%	12%	<1%	n.d.[c]
5	Kphth (1.0 equiv)	7%	2%	5%	5%	1%	n.d.[c]
6	DMAP (1.0 equiv)	18%	7%		2%	1%	n.d.[c]
7	$B(C_6F_5)_3$ (1.0 equiv)	<1%	n.d.[d]	4%	7%	d2%	n.d.[c]
8	MgI_2 (1.0 equiv)	7%	3%	5%	4%	0%	n.d.[c]
9	ZnI_2 (1.0 equiv)	7%	1%	5%	9%	<1%	n.d.[c]
9	$ZnCl_2$ (1.0 equiv)	14%	1%	5%	12%	<1%	n.d.[c]

[a] Based on GC integration relative to dodecane; [b] Based on ^{19}F NMR integrations of diagnostic resonances relative to that of fluorobenzene (–113.0 ppm) or α,α,α -trifluorotoluene (–62.6 ppm); [c] Not detected by GC or GC-MS [d] Observed by GC or GC-MS but not quantified due to poor NMR resolution

2.5 Evaluation of alternative electrophiles using Ni-1 as precatalyst

Scheme S2. General procedure for catalytic screens using **S8** or **S9** as the electrophile.

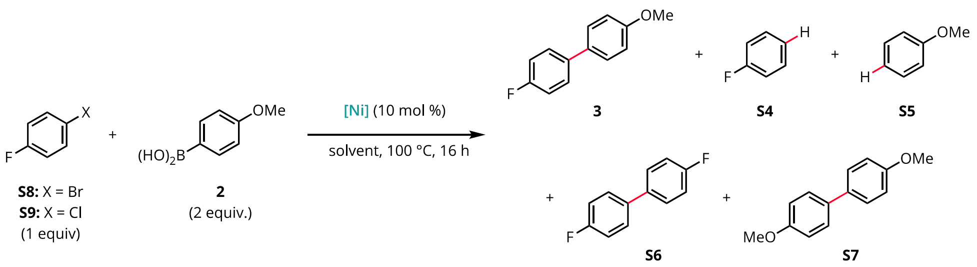

Table S4. Evaluation of alternative haloarene electrophiles using **Ni-1** as precatalyst

Entry	X=	Solvent (M)	Conv of **1** (%) [b]	Yield (%)				
				**3** [b]	**S4** [a]	**S5** [a]	**S6** [b]	**S7**
1	I (**1**)	THF (0.2 M)	15%	2%	16%	12%	<1%	n.d.[c]
2	I (**1**)	1,4-dioxane (0.2 M)	17%	2%	17%	11%	1%	n.d.[c]
3	I (**1**)	t-amyl alcohol (0.2 M)	18%	2%	12%	64%	<1%	n.d.[c]
4	Br (**S8**)	THF (0.2 M)	25%	<1%	13%	13%	<1%	n.d.[c]
5	Br (**S8**)	1,4-dioxane (0.2 M)	41%	1%	11%	9%	<1%	n.d.[c]
6	Br (**S8**)	t-amyl alcohol (0.2 M)	24%	<1%	6%	>99%	<1%	n.d.[c]
7	Cl (**S9**)	THF (0.2 M)	7%	n.d.[c]	1%	8%	3%	n.d.[c]
8	Cl (**S9**)	1,4-dioxane (0.2 M)	19%	n.d.[c]	3%	9%	n.d.[c]	n.d.[c]
9	Cl (**S9**)	t-amyl alcohol (0.2 M)	14%	n.d.[c]	2%	>99%	n.d.[c]	n.d.[c]

[a] Based on GC integration relative to dodecane; [b] Based on ^{19}F NMR integrations of diagnostic resonances relative to that of fluorobenzene (–113.0 ppm) or α,α,α -trifluorotoluene (–62.6 ppm); [c] Not detected by GC or GC-MS

Scheme S3. General procedure for catalytic screens using **S10** as the electrophile.

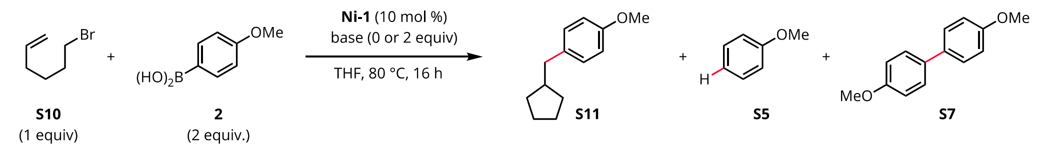

Table S5. Evaluation of C(sp^2)–C(sp^3) coupling using **Ni-1** as precatalyst

Entry	Base	Solvent (M)	Conversion of **S10** (%) [a]	Yield (%)		
				**S11** [b]	**S5** [a]	**S7**
1	none	THF (0.2 M)	73%	0%	9%	n.d.[c]
2	KO^{t}Bu	THF (0.2 M)	99%	1%	12%	n.d.[c]
3	KO^{t}Bu	PhMe (0.2 M)	99%	0%	17%	n.d.[c]
4	KO^{t}Bu	1,4-dioxane (0.2 M)	99%	2%	34%	n.d.[c]
5	KF	THF (0.2 M)	60%	trace	10%	n.d.[c]
6	K_3PO_4	THF (0.2 M)	85%	trace	10%	n.d.[c]
7	Na_2CO_3	THF (0.2 M)	70%	0%	9%	n.d.[c]

[a] Based on GC integration relative to dodecane; [b] Based on ^{1}H NMR integration of a diagnostic resonance (2.54 ppm) relative to that of dibromomethane (4.93 ppm)); [c] Not detected by GC or GC-MS

3. Synthesis and Characterization of Nickel Complexes

3.1 Oxidative Addition Product of Ni-2 and 2-Iodotoluene (Ni-3I)

In an N_2-filled glovebox, **Ni-2** (50.0 mg, 0.116 mmol, 1.00 equiv) was weighed into a scintillation vial. The scintillation vial was then charged with 2-iodotoluene (50.7 mg or 29.6 μL, 0.232 mmol, 2.00 equiv) and toluene (3 mL). The resulting yellowed orange solution was then stirred vigorously at room temperature for 1 h. The reaction mixture was then concentrated in vacuo to obtain crimson red solid. A monster pipette was loaded with a short pad of celite (approximately 2 cm height) and the celite was packed with pentane. The crimson red solid was then resuspended in pentane and passed through celite rinsing with total ~10 mL pentane total (colorless solution). The crimson red solid left on top of celite was further rinsed with total ~10 mL diether ether (light orange solution). The ether solution was discarded. The red solid was then rinsed with THF until colorless solution started eluting. The THF solution concentrated in vacuo to obtain crimson red solid in quantitative yield (0.0663 g).

To obtain single crystals suitable for XRD, the product was dissolved in minimum amount of THF in 1-dram vial. The 1-dram vial was placed in a scintillation vial containing pentane. The scintillation vial was sealed and left at room temperature.

^{1}H NMR (500 MHz, THF-d_8) δ 9.82 (d, J = 5.5 Hz, 1H), 8.15 (d, J = 2.2 Hz, 1H), 8.05 (t, J = 7.6 Hz, 1H), 7.79 (d, J = 8.1 Hz, 1H), 7.37 (t, J = 6.5 Hz, 1H), 6.97 (d, J = 2.1 Hz, 1H), 6.93 (d, J = 7.5 Hz, 1H), 6.57 (s, 1H), 6.48 (s, 1H), 6.16 (d, J = 7.3 Hz, 1H), 6.08 (t, J = 7.2 Hz, 1H), 5.97 (t, J = 7.5 Hz, 1H), 2.67 (s, 3H), 2.15 (s, 3H), 2.05 (s, 3H), 1.84 (s, 3H).

^{13}C NMR (126 MHz, THF-d_8) δ 175.4, 155.7, 151.57, 143.0, 142.7, 141.3, 138.8, 138.7, 136.2, 134.8, 134.2, 129.8, 129.6, 127.2, 126.0, 123.4, 122.6, 121.0, 116.7, 111.1, 35.2, 30.8, 28.7, 23.4, 21.1, 18.7, 18.3, 14.5

SC-XRD: The asymmetric unit contains one molecule in a general position. The Ni(iodido)(tolyl) portion of the molecule is modeled as disordered over two positions (0.54:0.46) that correspond to two orientations of the tolyl ligand.

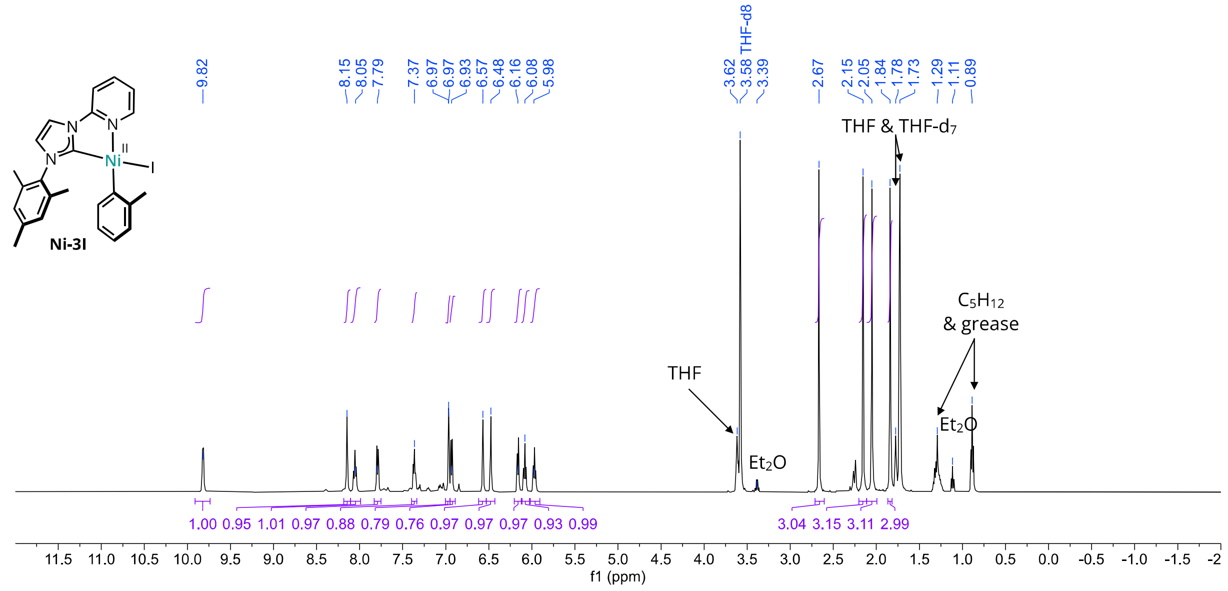

Figure S1. ^{1}H NMR (500 MHz, THF-d_8) spectrum of **Ni-3I**

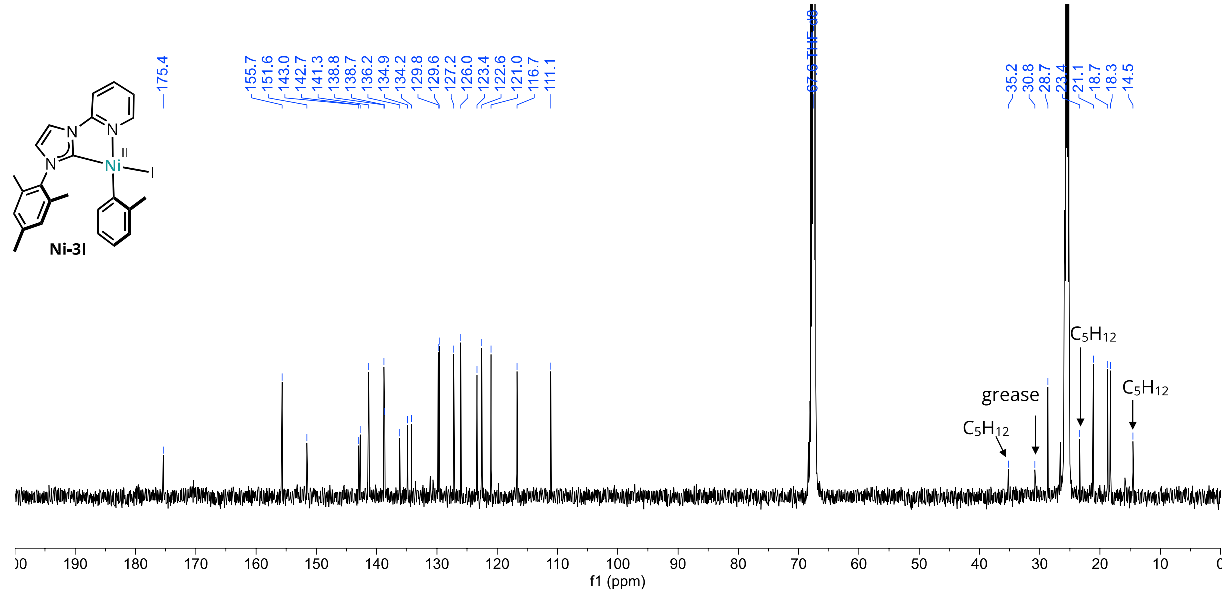

Figure S2. ^{13}C NMR (126 MHz, THF-d_8) spectrum of **Ni-3I**

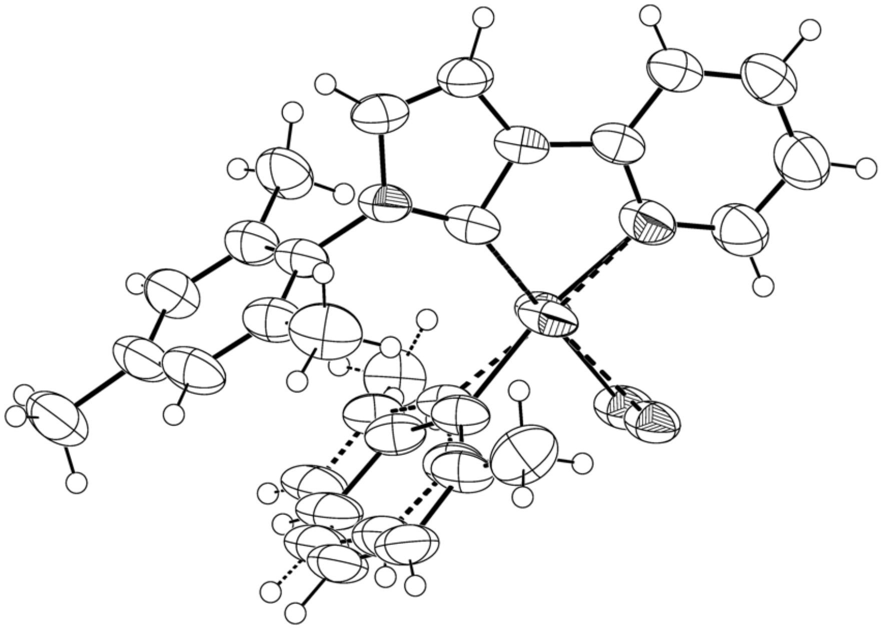

Figure S3. Asymmetric unit in the solid-state structure of **Ni-3l** determined by SC-XRD.

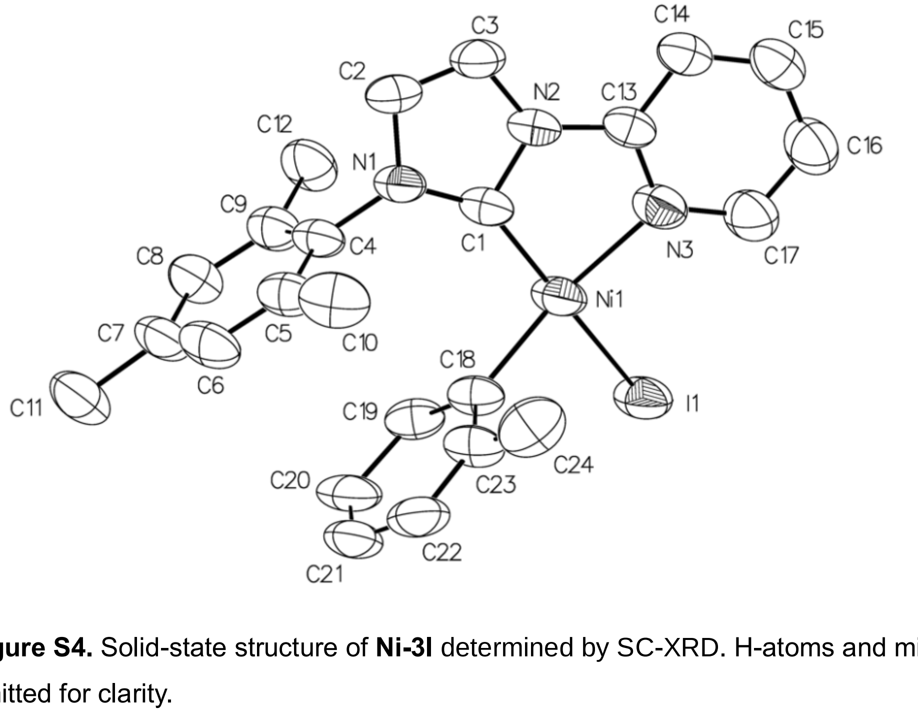

Figure S4. Solid-state structure of **Ni-3l** determined by SC-XRD. H-atoms and minor stereoisomer omitted for clarity.

3.2 Oxidative Addition Product of Ni-2 and 2-Bromotoluene (Ni-3Br)

In an N_2-filled glovebox, **Ni-2** (50.0 mg, 0.116 mmol, 1.00 equiv) was weighed into a scintillation vial. The scintillation vial was then charged with 2-bromotoluene (39.7 mg or 27.9 µL, 0.232 mmol, 2.00 equiv) and THF (3 mL). The resulting mixture was stirred vigorously at room temperature for 1 h. After 1 h, the reaction mixture was concentrated in vacuo. A monster pipette was loaded with a short pad of celite (approximately 2 cm height) and the celite was packed with pentane. The yellowed orange solid was then resuspended in pentane and passed through celite rinsing with total ~10 mL pentane total (colorless solution). The yellowed orange solid left on top of celite was further rinsed with total ~10 mL diether ether (light orange solution). The ether solution was discarded. The yellowed orange solid was then rinsed with THF until colorless solution started eluting. The THF solution concentrated in vacuo to obtain crimson red solid in 90% yield (0.0514 g).

To obtain single crystals suitable for XRD, the product was dissolved in minimum amount of THF, layered with pentane and left at room temperature. The supernatant was decanted, and the solid was dried in vacuo to obtain a metallic gold/yellow solid in 62% recrystallized yield (0.0356 g).

^{1}H NMR (500 MHz, CD_2Cl_2) δ 9.47 (d, *J* = 5.5 Hz, 1H), 8.03 (t, *J* = 7.8 Hz, 1H), 7.65 (s, 1H), 7.45 (d, *J* = 8.2 Hz, 1H), 7.39 (t, *J* = 6.6 Hz, 1H), 6.93 (d, *J* = 7.4 Hz, 1H), 6.73 (s, 1H), 6.55 (s, 1H), 6.49 (s, 1H), 6.32 – 6.20 (m, 1H), 6.28 (s, 1H), 6.14 (t, *J* = 7.4 Hz, 1H), 2.71 (s, 3H), 2.16 (s, 3H), 2.03 (s, 3H), 1.87 (s, 3H).

^{13}C NMR (126 MHz, CD_2Cl_2) δ 173.2, 151.5, 150.3, 144.0, 142.4, 140.8, 138.6, 136.6, 135.0, 134.2, 133.6, 129.1, 129.0, 126.6, 126.0, 122.8, 122.6, 121.1, 115.5, 109.8, 27.2, 20.9, 18.4, 18.0.

SC-XRD: The asymmetric unit contains one molecule in a general position. The tolyl ligand is modeled as disordered over two positions (0.62:0.38). The angle between the C1-Ni1-N3 and C18-Ni1-Br1 planes is 18.1(4) degrees; however, this value may be inaccurate due to the disorder, which likely extends into the metal center and beyond, although it was only modeled in the tolyl ligand due to resolution limitations.

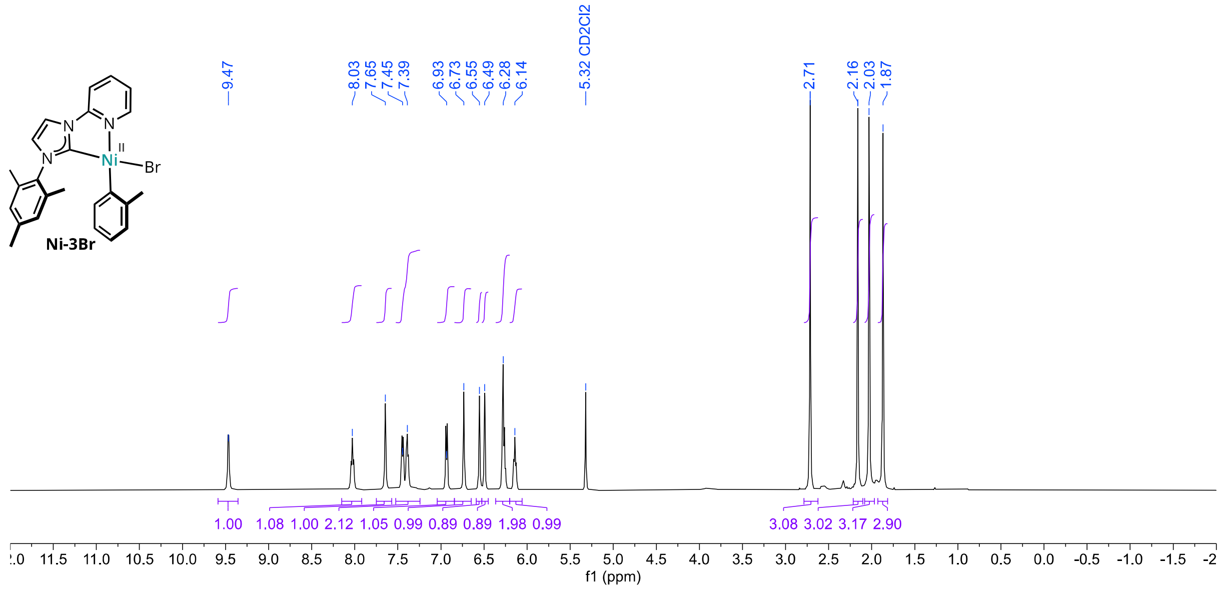

Figure S5. ^{1}H NMR (500 MHz, CD_2Cl_2) spectrum of **Ni-3Br**

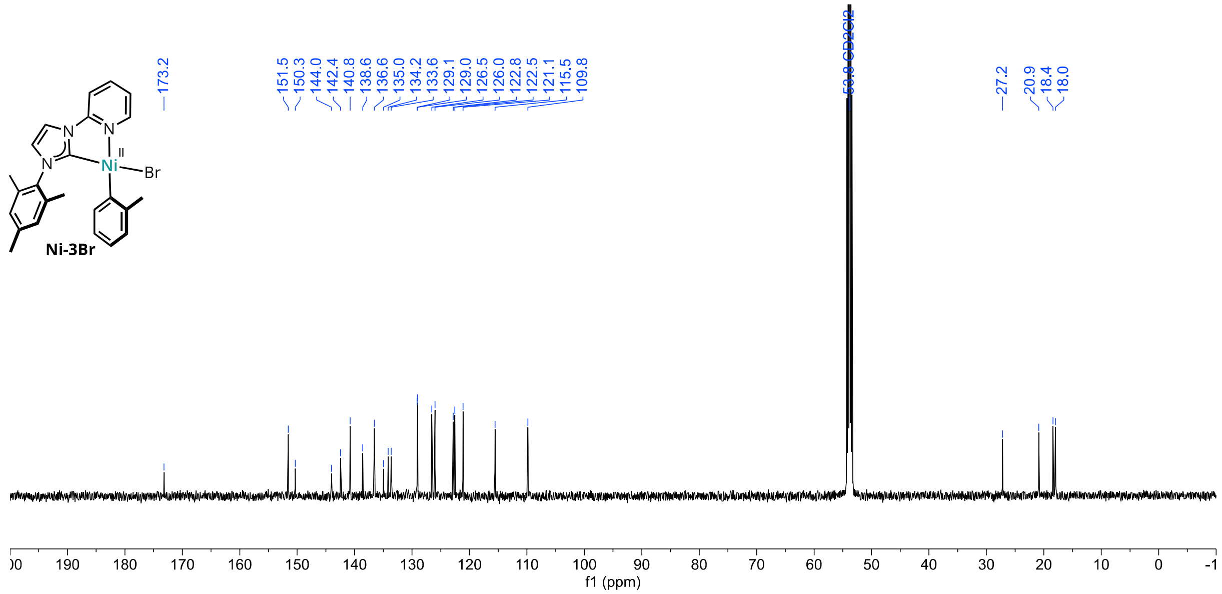

Figure S6. ^{13}C NMR (126 MHz, CD_2Cl_2) spectrum of **Ni-3Br**

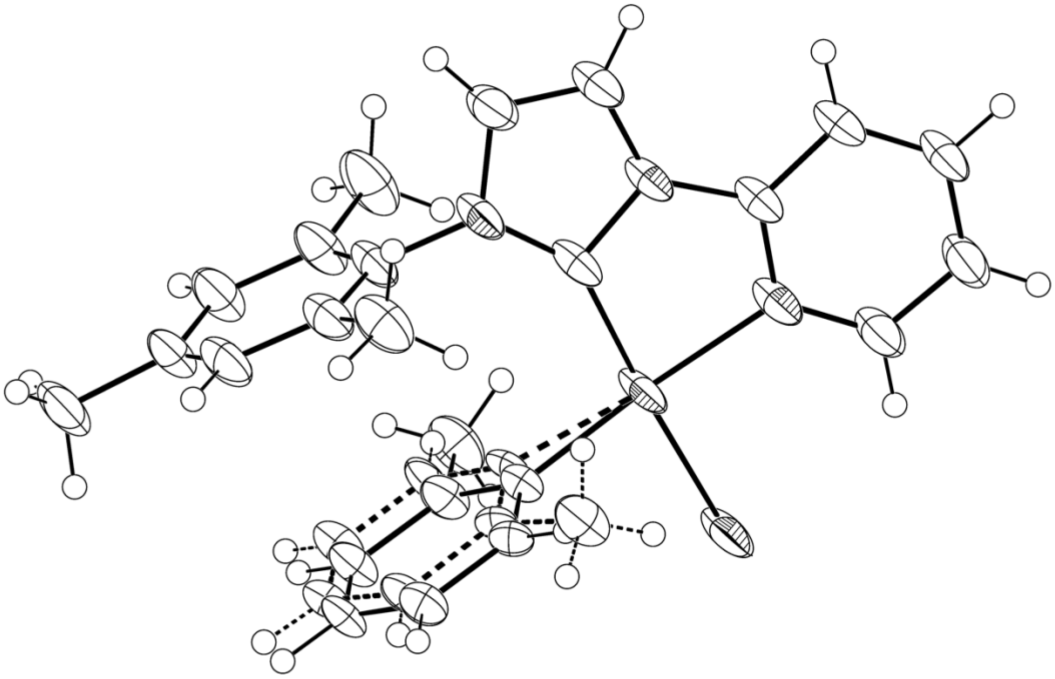

Figure S7. Asymmetric unit in the solid-state structure of **Ni-3Br** determined by SC-XRD.

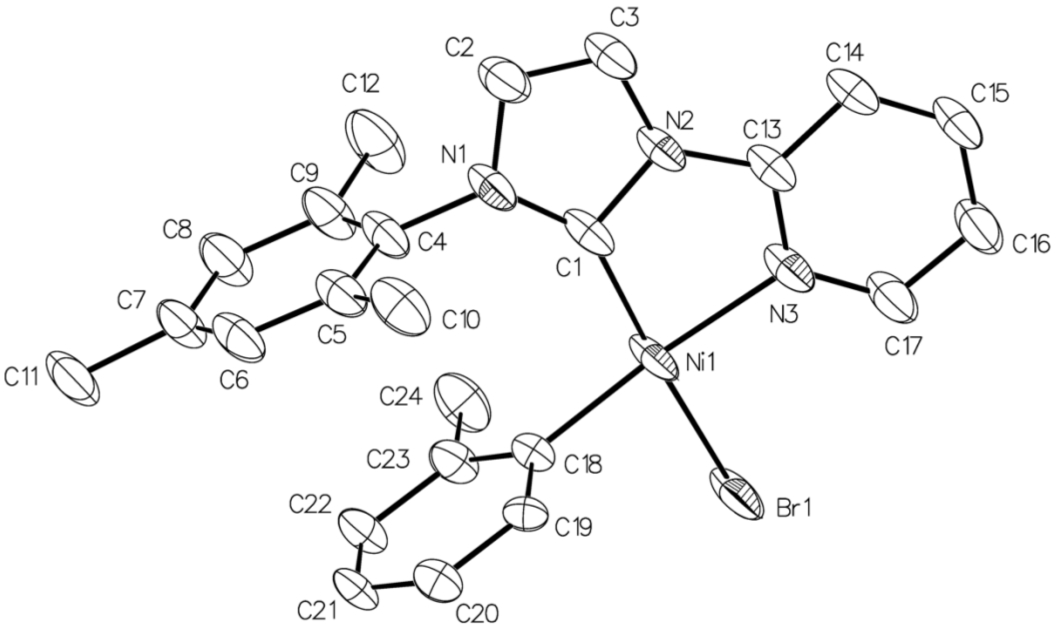

Figure S8. Solid-state structure of **Ni-3Br** determined by SC-XRD. H-atoms and minor stereoisomer omitted for clarity.

3.3 Oxidative Addition Product of Ni-1 and 2-halotoluene ([Ni-5]$_2$)

With 2-iodotoluene and 2-bromotoluene:

In an N_2-filled glovebox, [(^{h}IMesPyO)Ni(MeCN)][K(18-crown-6)] (0.0293 g, 0.043 mmol, 1.00 equiv) was weighed into a scintillation vial. C_6H_6 (0.02 M, 2.1 mL) was added into the vial, followed by 2-iodotoluene (10.9 µL, 0.0147 g, 0.086 mmol, 2.00 equiv) or 2-bromotoluene (10.3 µL, 0.0187 g, 0.086 mmol, 2.00 equiv). The resulting mixture was stirred vigorously at room temperature for 1 h. After 1 h, the yellowed orange turbid solution was loaded onto a monster pipette filled with a short pad of celite (approximately 2 cm height) and passed through celite rinsing with C_6H_6 until the filtrate was colorless. The filtrate was concentrated in vacuo and washed with pentane (2 × 2mL), decanting the pentane with a pipet after each wash. The solid was dried in vacuo to afford 20.8 mg of material.

To obtain single crystals suitable for XRD, the product was dissolved in minimum amount of THF, layered with pentane and left at room temperature.

With 2-chlorotoluene:

In an N_2-filled glovebox, [(^{h}IMesPyO)Ni(MeCN)][K(18-crown-6)] (0.0293 g, 0.043 mmol, 1.00 equiv) was weighed into a scintillation vial. C_6H_6 (0.02 M, 2.1 mL) was added into the vial, followed by 2-chlorotoluene (10.1 µL, 0.0109 g, 0.086 mmol, 2.00 equiv). The resulting mixture was stirred vigorously at room temperature for 1 h. After 1 h, the reaction mixture was concentrated in vacuo. Upon no **Ni-1** consumption as evidenced by ^{1}H NMR analysis of the crude mixture (C_6D_6, 400 MHz), the starting material was re-exposed to 2-chlorotoluene (10.1 µL, 10.9 mg, 0.086 mmol, 2.00 equiv) and transferred into a JY tube using C_6D_6 (0.03 M, 1.5 mL). The reaction mixture was heated at 50 ºC in a bead bath for 14 hours. After 14 hours, the reaction mixture was transferred into a 20 mL scintillation vial using THF (~3 mL) and concentrated in vacuo. A monster pipette was loaded with a short pad of celite (approximately 2 cm height) and the celite was packed with pentane. The yellowed orange solid was then resuspended in pentane and passed through celite rinsing with total ~5 mL pentane total (colorless solution). The pentane mixture was discarded. Into a separate vial the yellowed orange solid left on top of celite was further rinsed with total ~5 mL diethyl ether (light orange solution). The yellowed orange solid was then rinsed with benzene until colorless solution started eluting (~8 mL). Both the ether and benzene solutions were concentrated in vacuo to obtain a crimson red solid in 90% yield (0.0165 mg, 0.02 mmol).

^{1}H NMR (400 MHz, CD_3OD): δ 7.94 (s, 2H), 7.55 (dd, *J* = 8.6, 7.7 Hz, 2H), 7.10 (d, *J* = 7.7 Hz, 1H), 6.94 (s, 2H), 6.71 (d, *J* = 7.4 Hz, 2H), 6.54 (s, 2H), 6.47 (s, 2H), 6.43 – 6.33 (m, 3H), 6.29 (d, *J* = 8.6 Hz, 1H), 6.24 (dd, *J* = 7.7, 6.9 Hz, 2H), 2.87 (s, 6H), 2.11 (s, 6H), 2.02 (s, 6H), 1.88 (s, 6H).

^{1}H NMR (400 MHz, C_6D_6) δ 7.78 (d, *J* = 2.0 Hz, 2H), 7.39 (t, *J* = 8.0 Hz, 2H), 6.94 (d, *J* = 7.4 Hz, 2H), 6.82 – 6.73 (m, 2H), 6.55 (d, *J* = 7.5 Hz, 2H), 6.37 (s, 2H), 6.31 (s, 2H), 6.28 – 6.19 (m, 2H), 6.22 (s, 2H), 6.13 (d, *J* = 8.5 Hz, 2H), 6.08 (t, *J* = 7.3 Hz, 2H), 2.75 – 2.65 (m, 6H), 1.96 (s, 6H), 1.86 (s, 6H), 1.72 (s, 6H).

13C NMR spectra were not obtained due to poor solubility of the sample.

SC-XRD: The asymmetric unit contains one-half of a dinickel complex located adjacent to a crystallographic two-fold axis and one deuterated benzene solvent molecule in a general position. The toluenyl ligand is modeled as disordered over two positions (0.82:0.18). The angle between the C1-Ni1-N3 and C18-Ni1-O1[symmetry equivalent] planes is 21.13(14) degrees.

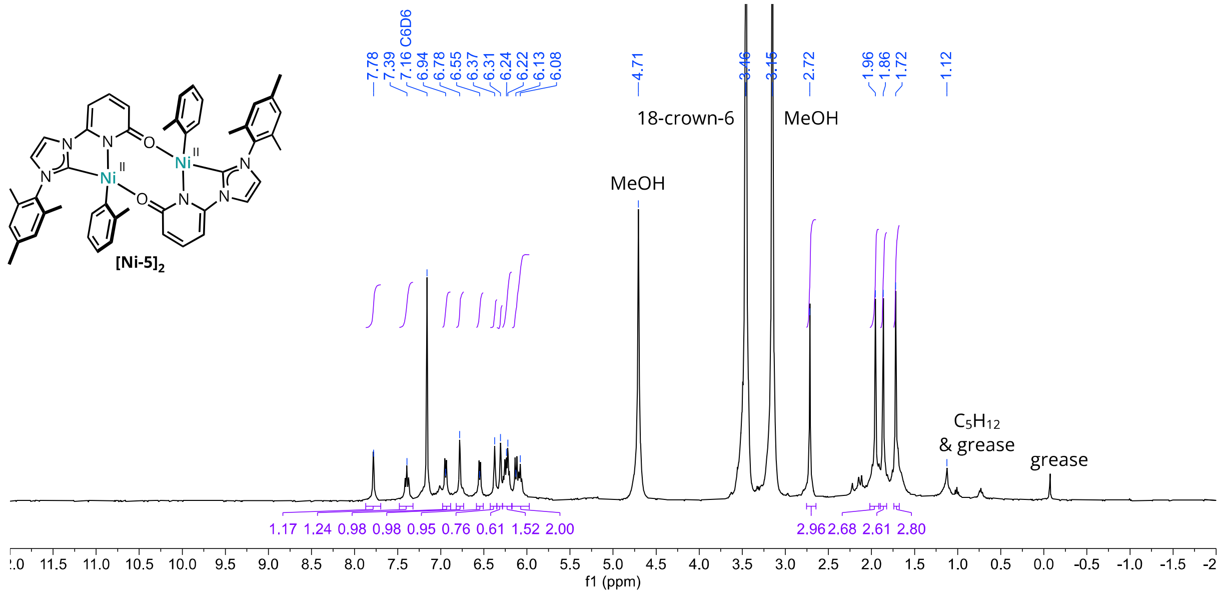

Figure S9. ^{1}H NMR (500 MHz, C_6D_6) spectrum of **[Ni-5]**$_2$

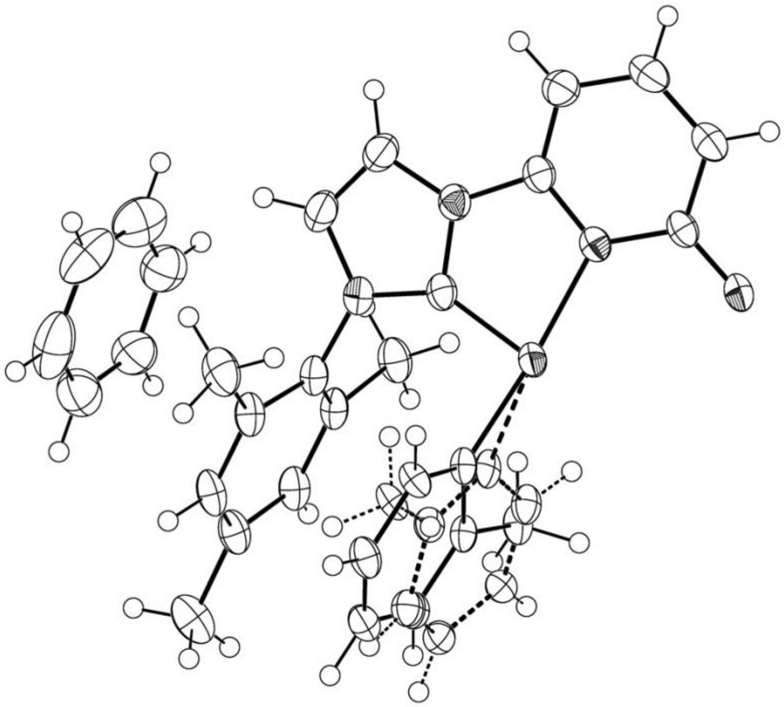

Figure S10. Asymmetric unit in the solid-state structure of **[Ni-5]$_2$** determined by SC-XRD.

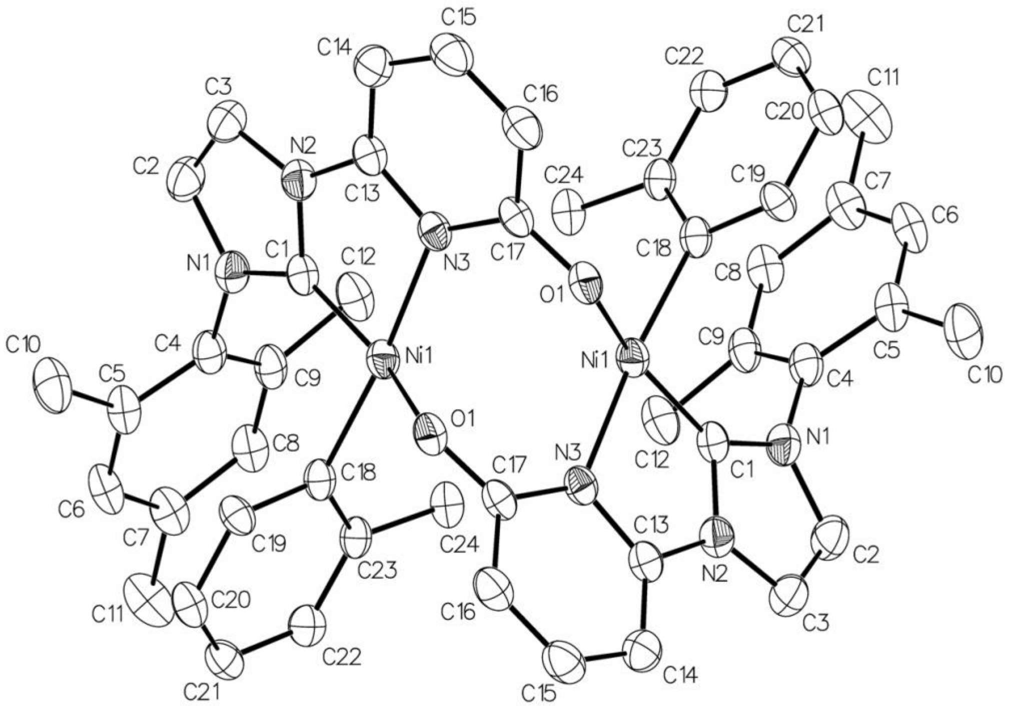

Figure S11. Solid-state structure of **[Ni-5]$_2$** determined by SC-XRD. H-atoms and solvent molecules omitted for clarity

3.4 Oxidative Addition Product of Ni-1 and 4-Fluoroiodobenzene

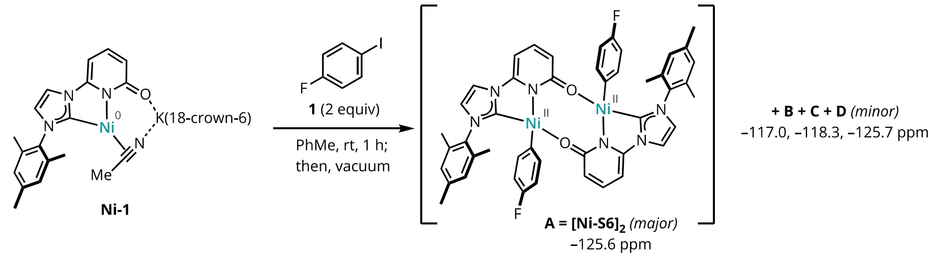

In an N_2-filled glovebox, [(^{h}IMesPyO)Ni(MeCN)][K(18-crown-6)] (**Ni-1**, 0.014 g, 0.020 mmol, 1 equiv) and 2-iodotoluene (0.009 g, 0.04 mmol, 2 equiv) were weighed into a scintillation vial. A PTFE-coated magnetic stir bar and toluene (0.01 M, 2 mL) were added into the vial. The vial was sealed, and the resulting mixture was stirred vigorously at room temperature for 1 h. After 1 h, the solvent was removed in vacuo from yellowed orange turbid solution. The resulting yellowed orange solid was washed with pentane (3 × 2 mL), decanting each wash with a pipette. The solid was dried in vacuo and used in crude form in subsequent steps. Upon resuspending the solid in methanol-d_4, ^{1}H and ^{19}F NMR spectroscopic features were found to be consistent with a single major (^{19}F NMR δ: –125.6 ppm) and three minor (^{19}F NMR δ: –117.0, –118.3, and –125.7 ppm) fluorophenyl-containing products. Based on comparison of the ^{1}H NMR spectrum of the crude product with those of [**Ni-5**]$_2$ and **Ni-3I**, an analogous dimeric structure **[Ni-S6]$_2$** is proposed to be the major component. Attempts to obtain single crystals suitable for XRD were unsuccessful

^{1}H NMR (500 MHz, CD_3OD): δ 7.95 (d, *J* = 2.3 Hz, 1H), 7.69 (t, *J* = 6.7 Hz, 1H), 7.55 (t, *J* = 7.9 Hz, 1H), 7.23–7.06 (m, 2H), 6.93 (s, 1H), 6.70 (d, *J* = 7.3 Hz, 1H), 6.55 (s, 2H), 6.26 (t, *J* = 9.5 Hz, 2H), 2.15 (s, 3H), 1.97 (s, 6H) apparent major component peaks only

^{19}F NMR (376 MHz, CD_3OD) δ –100.18, –108.29, –116.97, –118.30, –125.55, –125.67 mixture of components.

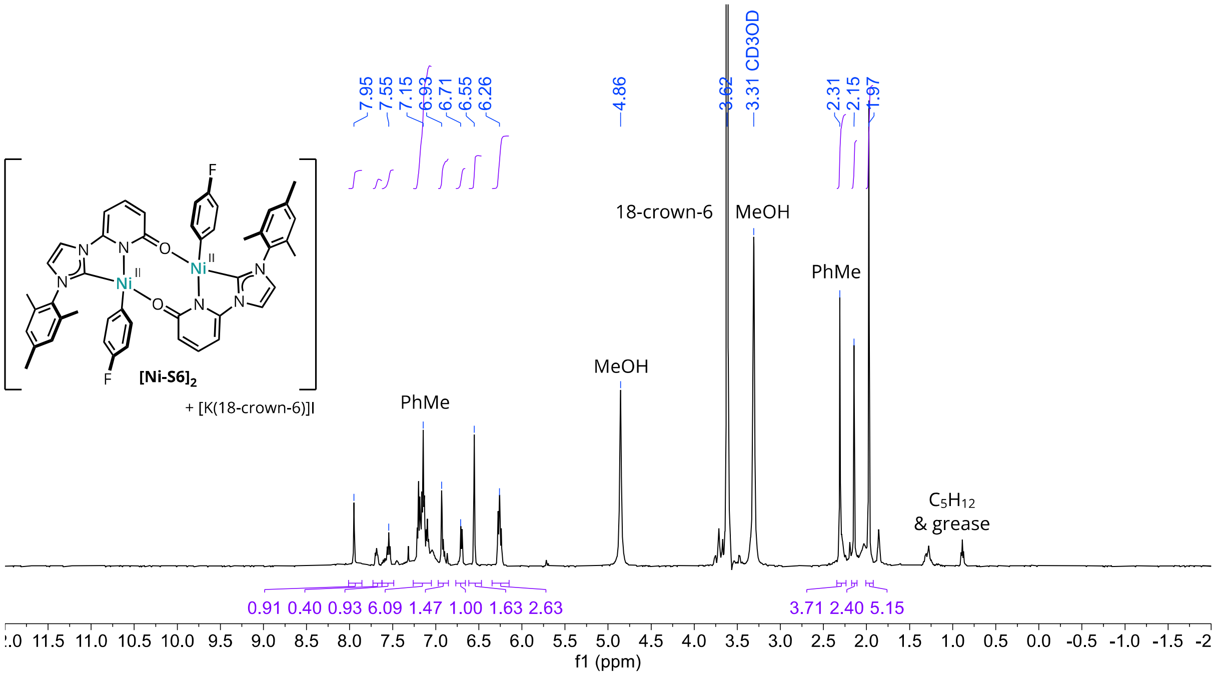

Figure S12. ^{1}H NMR (500 MHz, CD_3OD) spectrum of the product obtained following the reaction of **Ni-1** and 4-fluoroiodotoluene (**1**). Major component tentatively assigned as **[Ni-S6]₂**.

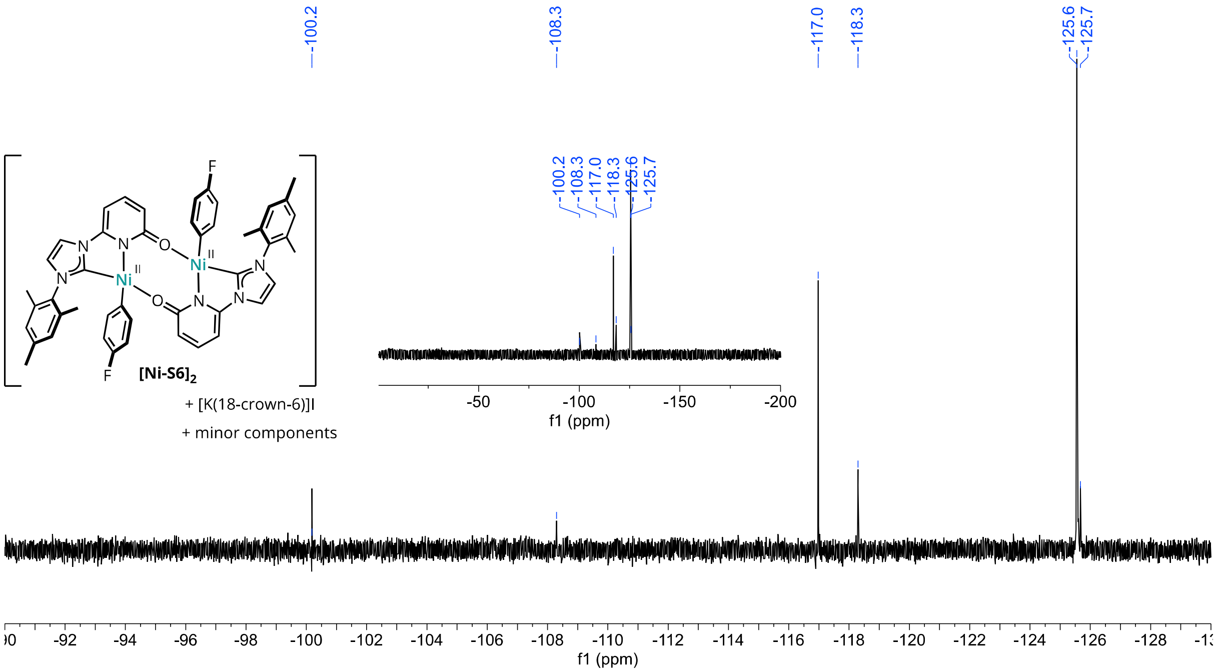

Figure S13. ^{19}F NMR (376 MHz, CD3OD) spectrum of the product obtained following the reaction of **Ni-1** and 4-fluoroiodotoluene (**1**). Major component tentatively assigned as **[Ni-S6]₂**.

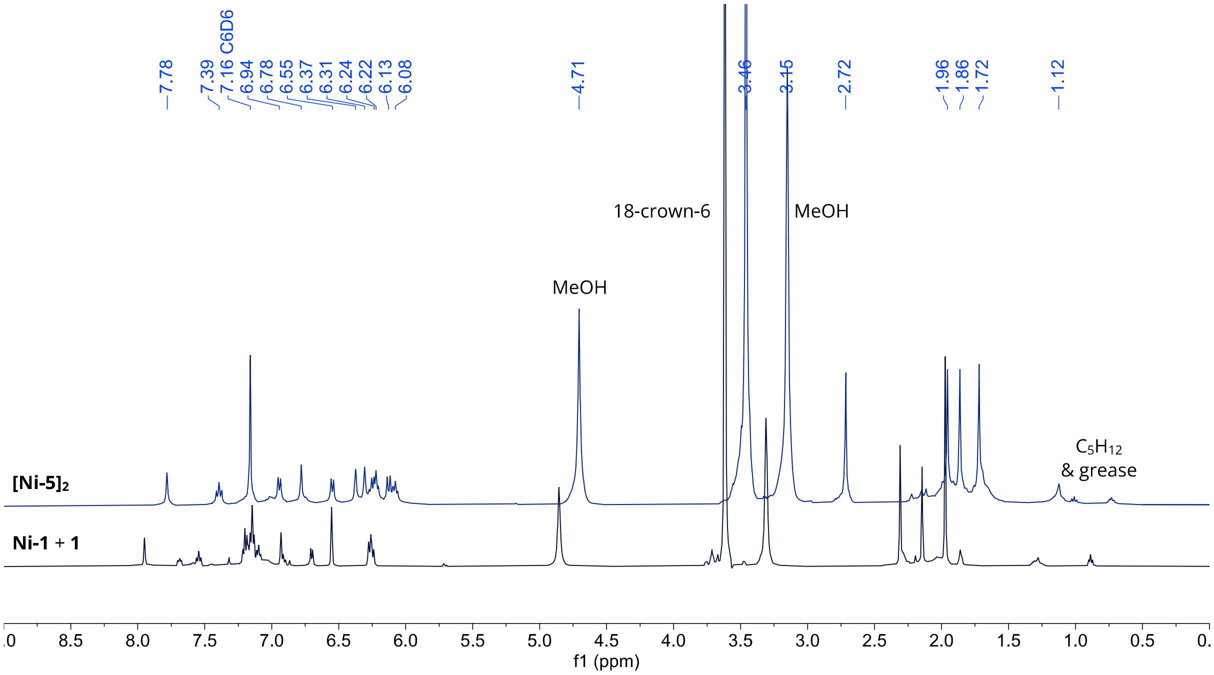

Figure S14. Comparison of the ^{1}H NMR spectra of **[Ni-5]$_2$** (500 MHz, C_6D_6 with added MeOH), and the product obtained following the reaction of **Ni-1** and 4-fluoroiodotoluene (**1**, 500 MHz, CD_3OD).

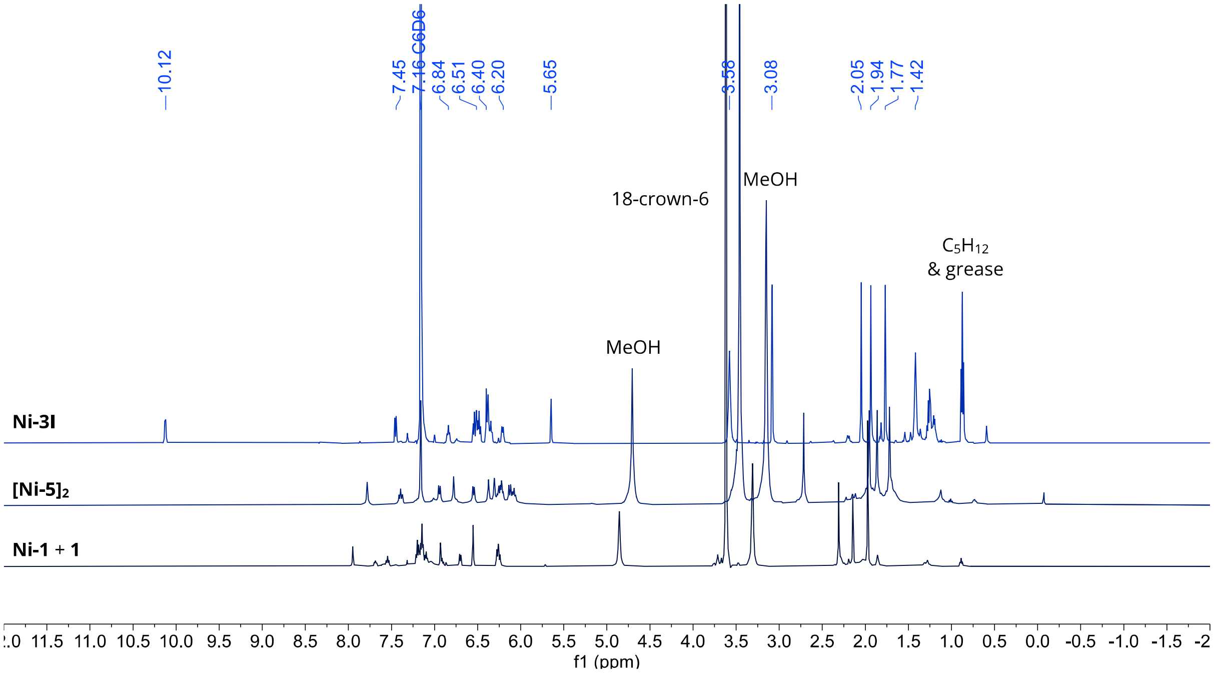

Figure S15. Comparison of the ^{1}H NMR spectra of **Ni-3I** (500 MHz, C_6D_6), **[Ni-5]$_2$** (500 MHz, C_6D_6 with added MeOH), and the product obtained following the reaction of **Ni-1** and 4-fluoroiodotoluene (**1**, 500 MHz, CD_3OD).

3.5 Summary of Crystallographic Data and Refinement Details

Table S6. Crystallographic data and refinement details

	**Ni-3I**	**Ni-3Br**	**$[Ni\text{-}5]_2$**
CCDC	2359151	2359153	2359152
Empirical formula	C24 H24 I N3 Ni	C24 H24 Br N3 Ni	C60 H46 D12 N6 Ni2 O2
Formula weight	540.07	493.08	1024.61
Crystal system	triclinic	triclinic	monoclinic
Space group	*P-1*	*P-1*	*I2/a*
Unit cell dimensions (Å or °)			
a	8.0858(2)	7.9257(2)	19.4741(3)
b	9.3111(2)	9.0321(2)	8.81630(10)
c	15.7060(4)	16.2254(5)	29.1312(3)
α	89.808(2)°	88.550(2)°	90°
β	87.396(2)°	89.695(2)°	100.3570(10)°
γ	70.291(2)°	66.645(3)°	90°
Volume ($Å^3$)	1111.96(5)	1065.98(5)	4920.03(11)
Z	2	2	4
Calculated density (Mg/m^3)	1.613	1.536	1.383
Absorption coefficient (mm^{-1})	12.258	3.612	1.349
F(000)	540	504	2128
Crystal color, morphology	orange, needle	orange, needle	yellow-orange, plate
Crystal size (mm^3)	0.095 × 0.057 × 0.022	0.096 × 0.06 × 0.047	0.168 × 0.115 × 0.052
θ range for data collection (°)	5.046 to 80.285	5.336 to 79.858	4.616 to 80.178
Index ranges	$-9 \leq h \leq 10$	$-10 \leq h \leq 10$	$-24 \leq h \leq 24$
	$-11 \leq k \leq 11$	$-11 \leq k \leq 11$	$-11 \leq k \leq 11$
	$-20 \leq l \leq 19$	$-20 \leq l \leq 20$	$-37 \leq l \leq 32$
Reflections collected	26694	34787	42162
Independent reflections	4716 [*R*(int) = 0.0454]	4547 [*R*(int) = 0.0415]	5303 [*R*(int) = 0.0602]
Observed reflections	3919	3970	4654
Completeness to θ	99.9%	99.5%	99.8%
Data / restraints / parameters	4716 / 103 / 349	4547 / 82 / 331	5303 / 70 / 385
Goodness-of-fit on F^2	1.063	1.044	1.077
Final *R* indices [I>2 σ(I)]	R_1 = 0.0489, wR_2 = 0.1219	R_1 = 0.0421, wR_2 = 0.0938	R_1 = 0.0485, wR_2 = 0.1115
R indices (all data)	R_1 = 0.0567, wR_2 = 0.1262	R_1 = 0.0481, wR_2 = 0.0971	R_1 = 0.0554, wR_2 = 0.1148
Largest diff. peak and hole (e.$Å^{-3}$)	0.874 and –0.856	1.069 and –0.902	0.758 and –0.525

4. Mechanistic Experiments

4.1 ^{19}F NMR Monitoring in Methanol-d_4

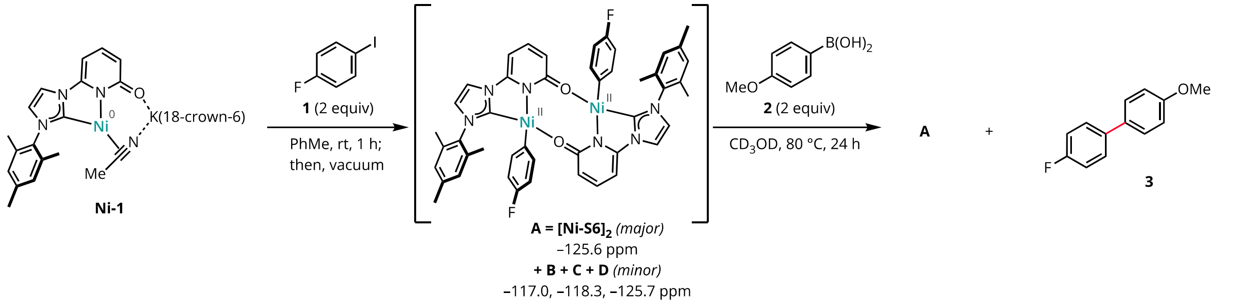

In an N_2-atmosphere glovebox, the crude precipitate obtained from the reaction of **Ni-1** and 4-fluoroiodotoluene (**1**, see Section 3.4) was resuspended in methanol-d_4 (1 mL) and transferred to a JYoung NMR tube. ^{1}H and ^{19}F NMR spectra were obtained, revealing features consistent with a single major (^{19}F NMR δ: –125.6 ppm, **A**) and three minor (^{19}F NMR δ: –117.0 (**B**), –118.3 (**C**), and –125.7 ppm (**D**)) fluorophenyl-containing products. The NMR tube was returned to the glovebox, and 4-methoxyphenylboronic acid (**2**, 0.006 g, 0.04 mmol, 2 equiv) was added as a solid, rinsing with minimal methanol-d_4 to quantitate the transfer. The tube was sealed, removed from the glovebox, and heated to 80 °C in a bead bath. ^{1}H and ^{19}F NMR spectra were obtained intermittently while heating to 80 °C over the course of 24 hours. The three minor species were largely consumed, as evidenced by the disappearance or decreased magnitude of the ^{19}F NMR resonances at –117.0, –118.3, and –125.7 ppm, coincident with the appearance of a new signature at –115.6 ppm (corresponding to product **3**). However, the resonance at –125.6 ppm (**A**), which was tentatively assigned as dimer **[Ni-S6]$_2$** persisted, even after heating to 80 °C for 24 hours.

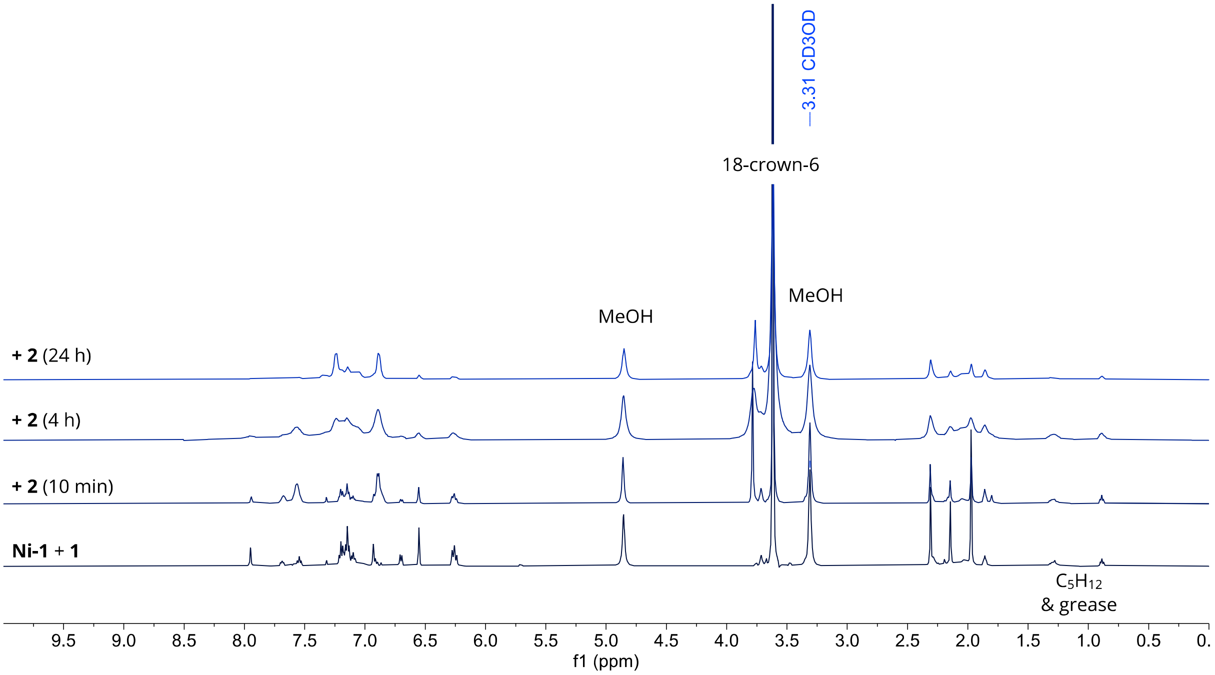

Figure S16. ^{1}H NMR (500 MHz, CD_3OD) spectra obtained over time upon treating crude **[Ni-6]$_2$** with **2** and heating to 80 °C.

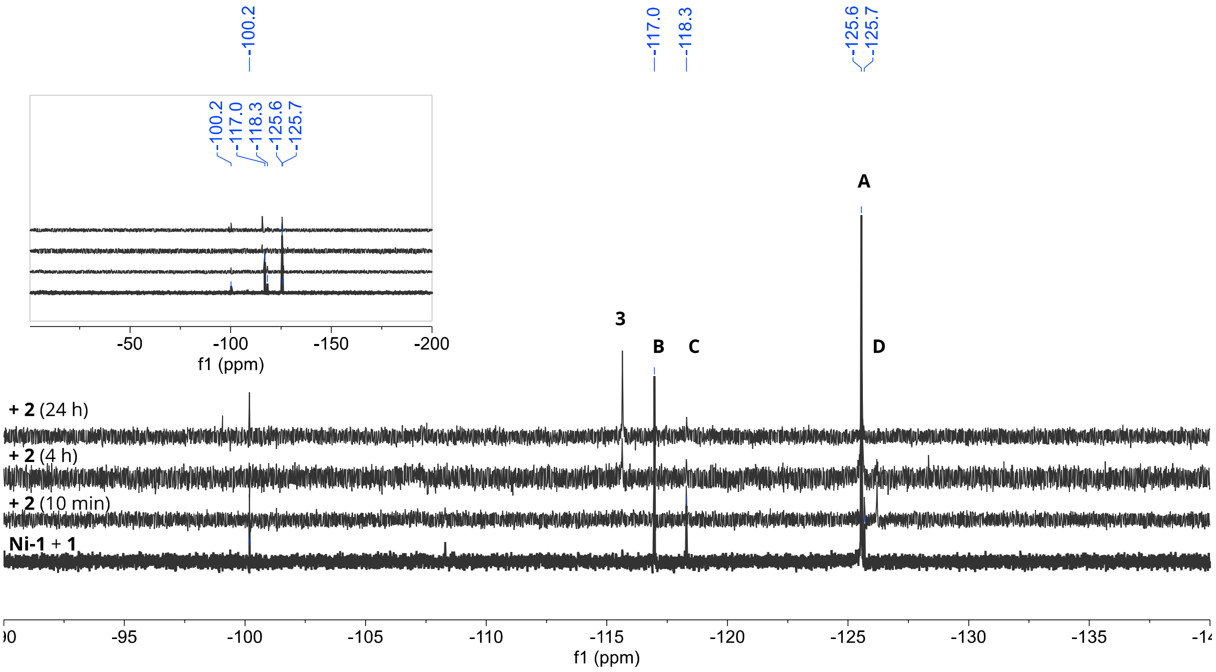

Figure S17. ^{19}F NMR (376 MHz, CD_3OD) spectra obtained over time upon treating crude **[Ni-6]$_2$** with **2** and heating to 80 °C.

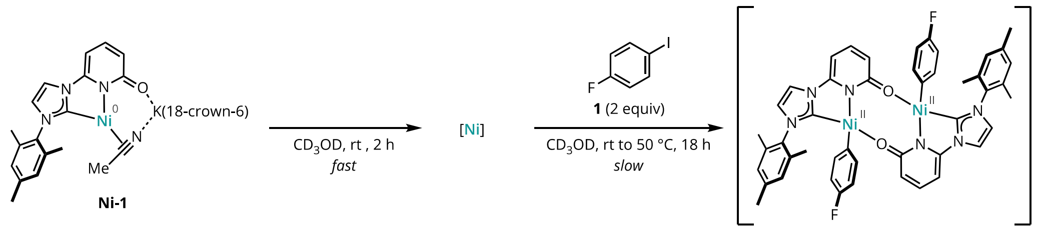

An analogous experiment was performed, with the goal of monitoring initial formation of **[Ni-6]₂** in CD_3OD. In an N_2-filled glovebox, [(^{h}IMesPyO)Ni(MeCN)][K(18-crown-6)] (**Ni-1**, 0.018 g, 0.027 mmol, 1.0 equiv) was weighed into a scintillation vial. Methanol-*d₄* (1 mL) was added, resulting in an immediate color change from carmine red to poppy red, and the resulting solution was transferred to a JYoung NMR tube. The ^{1}H NMR spectrum was obtained, revealing a decrease in symmetry compared to **Ni-1**. The NMR tube was returned to the glovebox, and 4-fluoroiodotoluene (**1**, 6.2 µL, 0.054 mmol, 2.0 equiv) was added. The tube was sealed, removed from the glovebox, and maintained at room temperature (~22 °C) while mixing on a rotary shaker. ^{1}H and ^{19}F NMR spectra were obtained after 1.5 hours but revealed negligible consumption of **1**. The NMR tube was then immersed in a bead bath to heat to 50°C. ^{1}H and ^{19}F NMR spectra were obtained intermittently while heating to 50 °C over the course of 15 hours. After 15 hours, partial conversion of **1** was observed, and a mixture of fluorophenyl-containing products including **A** (tentatively assigned as dimer **[Ni-S6]$_2$**) and fluorobenzene (arising from protodehalogenation of **1**) was formed. However, the initial reaction between the Ni(0) source (**Ni-1**) and the solvent appeared to inhibit productive reaction with the aryl halide (**1**).

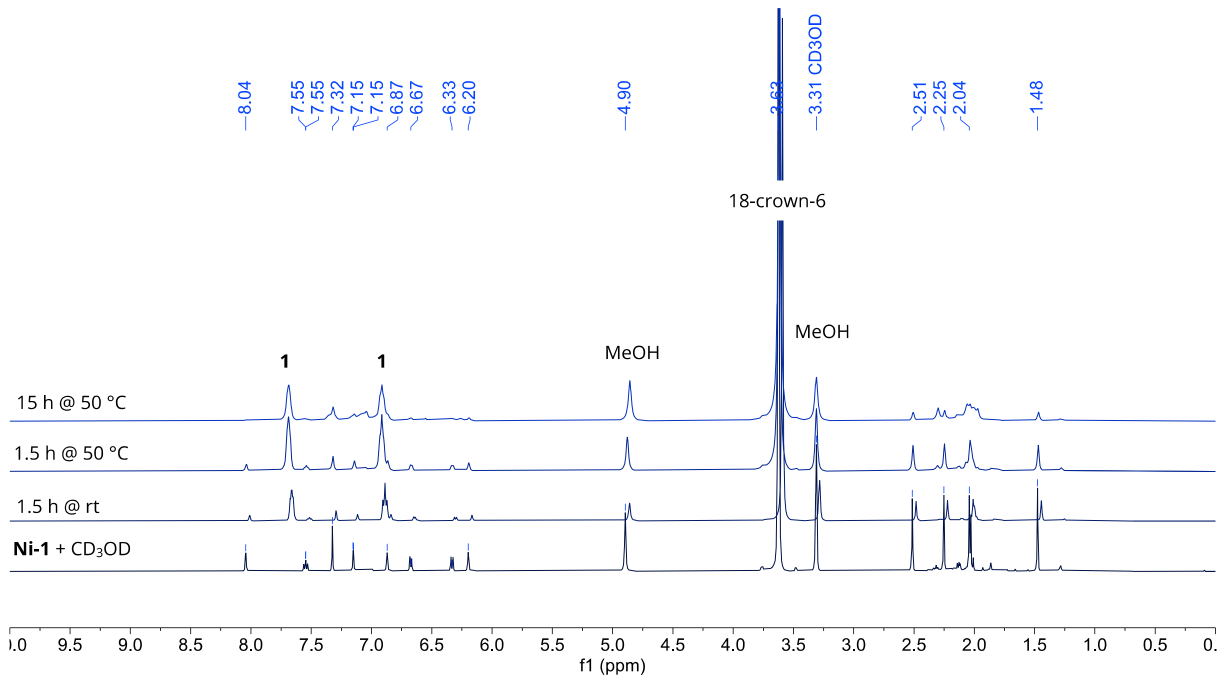

Figure S18. ^{1}H NMR (500 MHz, CD_3OD) spectra obtained over time upon treating **Ni-1** with **1** in CD_3OD and heating to 50 °C.

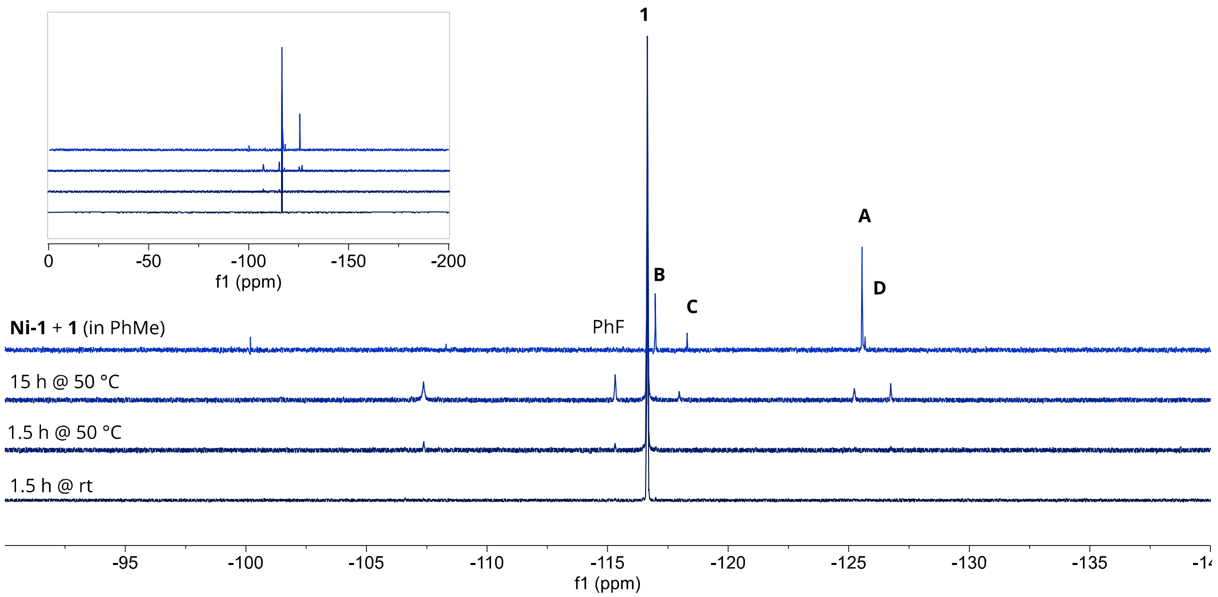

Figure S19. ^{19}F NMR (376 MHz, CD_3OD) spectra obtained over time upon treating **Ni-1** with **1** in CD_3OD and heating to 50 °C compared with the product mixture obtained upon reaction with **Ni-1** with **1** in PhMe.

4.2 ^{19}F NMR Monitoring in THF-d_8

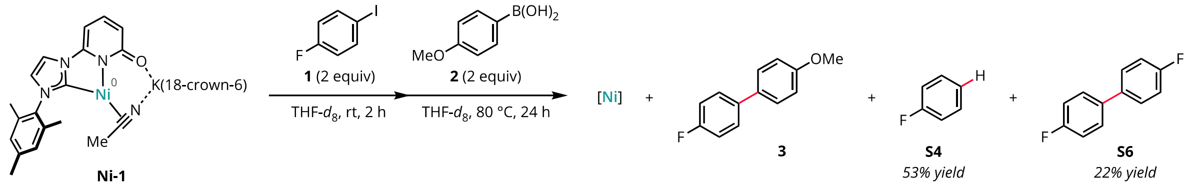

In an N_2-filled glovebox, [(^{h}IMesPyO)Ni(MeCN)][K(18-crown-6)] (**Ni-1**, 0.018 g, 0.027 mmol, 1.0 equiv) was weighed into a 1-dram vial. THF-d_8 (1 mL) was added, and the resulting solution was transferred to a JYoung NMR tube to obtain an initial ^{1}H NMR spectrum. The NMR tube was returned to the glovebox, and 4-fluoroiodotoluene (**1**, 6.2 µL, 0.054 mmol, 2.0 equiv) was added. The tube was sealed, removed from the glovebox, and maintained at room temperature (~22 °C) while mixing on a rotary shaker for 2 hours. ^{1}H and ^{19}F NMR spectra were collected. The NMR tube was returned to the glovebox, and 4-methoxyphenylboronic acid (**2**, 0.008 g, 0.054 mmol, 2.0 equiv) was added as a solid. The tube was sealed, removed from the glovebox, and immersed in a bead bath to heat to 80°C. ^{1}H and ^{19}F NMR spectra were obtained intermittently while heating to 80 °C over the course of 24 hours. After 24 hours, the NMR tube was returned to the glovebox, and α,α,α-trifluorotoluene (15 µL, 0.083 mmol, 3.1 equiv) was added as an internal standard for a final ^{19}F NMR spectrum. The NMR tube was then opened outside the glovebox, and dodecane (20 µL, 0.088 mmol, 3.26 equiv) was added as an internal standard for GC analysis of the organic products.

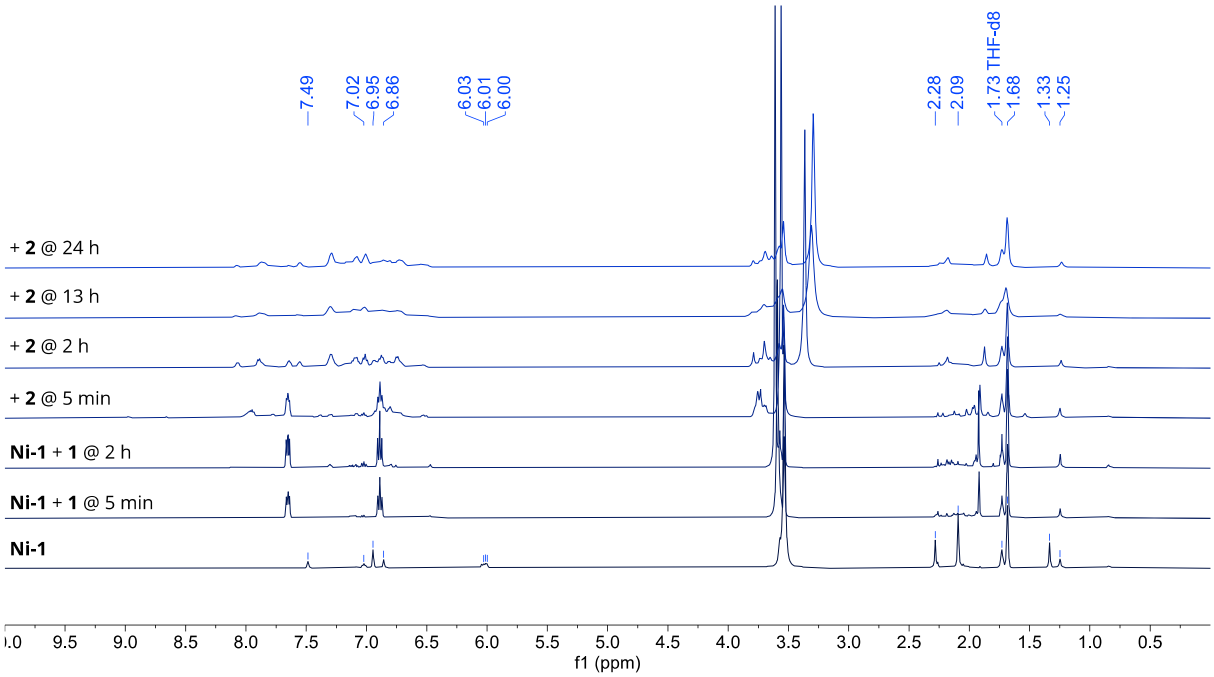

Figure S20. ^{1}H NMR (500 MHz, THF-d_8) spectra obtained over time upon treating **Ni-1** with **1** and **2** and heating to 80 °C.

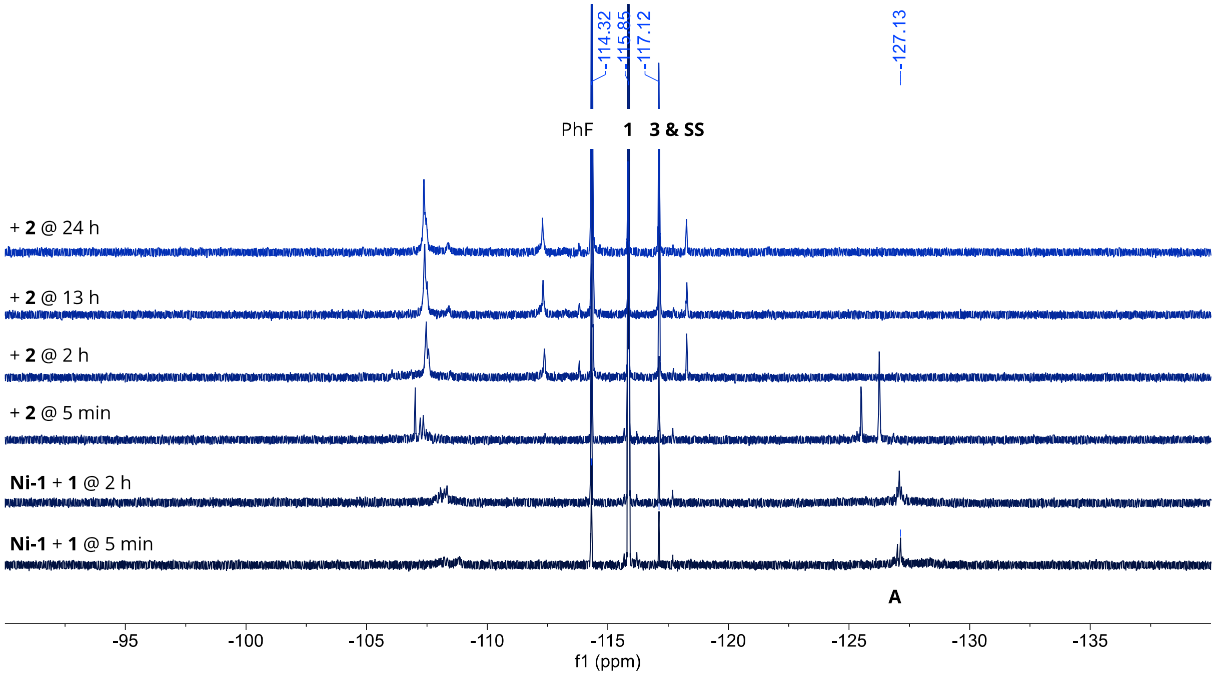

Figure S21. ^{19}F NMR (376 MHz, THF-d_8) spectra obtained over time upon treating **Ni-1** with **1** and **2** and heating to 80 °C.

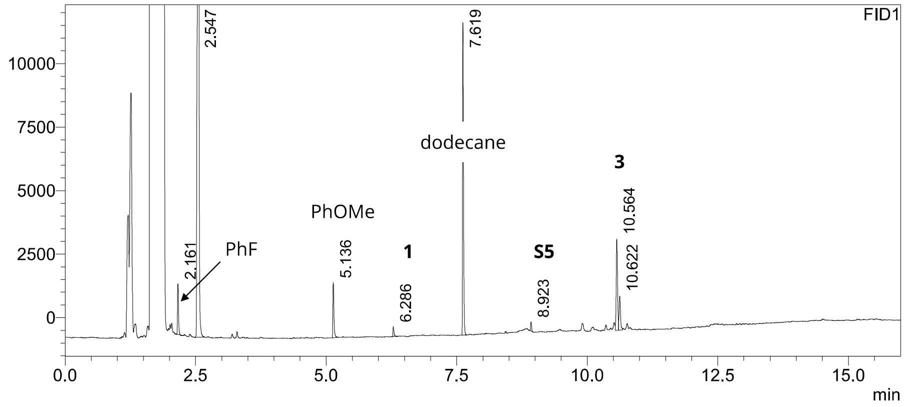

Peak#	Ret. Time	Area
1	2.161	2443
2	2.547	60837
3	5.136	3044
4	6.286	529
5	7.619	15130
6	8.923	440
7	10.564	6457
8	10.622	2516
Total		91396

Figure S22. FID-GC trace of the crude materials obtained upon treating **Ni-1** with **1** and **2** and heating to 80 °C.

6. References

(1) Afandiyeva, M.; Kadam, A. A.; Wu, X.; Brennessel, W. W.; Kennedy, C. R. Synthesis, Structure, and Hydroboration Reactivity of Anionic Nickel(0) Complexes Supported by Bidentate NHC-Pyridone Ligands. *Organometallics* **2022**, *41* (21), 3014-3023. DOI: 10.1021/acs.organomet.2c00439.

(2) Craig, S. M.; Malyk, K. R.; Silk, E. S.; Nakamura, D. T.; Brennessel, W. W.; Kennedy, C. R. Synthesis and characterization of Ni(0) complexes supported by an unsymmetric C,N ligand. *J. Coord. Chem.* **2022**, *75* (11-14), 1841-1852. DOI: 10.1080/00958972.2022.2117037.

(3) Pangborn, A. B.; Giardello, M. A.; Grubbs, R. H.; Rosen, R. K.; Timmers, F. J. Safe and Convenient Procedure for Solvent Purification. *Organometallics* **1996**, *15* (5), 1518–1520. DOI: 10.1021/om9503712.

(4) Fulmer, G. R.; Miller, A. J. M.; Sherden, N. H.; Gottlieb, H. E.; Nudelman, A.; Stoltz, B. M.; Bercaw, J. E.; Goldberg, K. I. NMR Chemical Shifts of Trace Impurities: Common Laboratory Solvents, Organics, and Gases in Deuterated Solvents Relevant to the Organometallic Chemist. *Organometallics* **2010**, *29* (9), 2176-2179. DOI: 10.1021/om100106e.

(5) Rosenau, C. P.; Jelier, B. J.; Gossert, A. D.; Togni, A. Exposing the Origins of Irreproducibility in Fluorine NMR Spectroscopy. *Angew. Chem. Int. Ed.* **2018**, *57* (30), 9528–9533. DOI: 10.1002/anie.201802620.

(6) *CrysAlisPro, version 171.42.57a*; Rigaku Corporation: Oxford, UK, 2022. (accessed.

(7) Sheldrick, G. SHELXT - Integrated space-group and crystal-structure determination. *Acta Crystallogr. A* **2015**, *71* (1), 3-8. DOI: doi:10.1107/S2053273314026370.

(8) Sheldrick, G. Crystal structure refinement with SHELXL. *Acta Crystallogr. C* **2015**, *71* (1), 3-8. DOI: 10.1107/S2053229614024218.

(9) Dolomanov, O. V.; Bourhis, L. J.; Gildea, R. J.; Howard, J. A. K.; Puschmann, H. OLEX2: a complete structure solution, refinement and analysis program. *J. Appl. Crystallogr.* **2009**, *42* (2), 339–341. DOI: 10.1107/S0021889808042726.

(10) Macrae, C. F.; Sovago, I.; Cottrell, S. J.; Galek, P. T. A.; McCabe, P.; Pidcock, E.; Platings, M.; Shields, G. P.; Stevens, J. S.; Towler, M.; et al. Mercury 4.0: from visualization to analysis, design and prediction. *J. Appl. Cryst.* **2020**, *53* (1), 226-235. DOI: 10.1107/S1600576719014092.
